# Complex drought patterns robustly explain global yield loss for major crops

**DOI:** 10.1038/s41598-022-09611-0

**Published:** 2022-04-06

**Authors:** Monia Santini, Sergio Noce, Marta Antonelli, Luca Caporaso

**Affiliations:** 1 Impacts on Agriculture, Forests and Ecosystem Services (IAFES) Division, Foundation Euro-Mediterranean Center on Climate Change (CMCC), 01100 Viterbo, Italy; 2 Barilla Center for Food & Nutrition (BCFN) Foundation, 43121 Parma, Italy; 3 European Commission - Joint Research Centre (JRC), 21027 Ispra, Italy

**Keywords:** Plant sciences, Climate sciences, Hydrology

## Abstract

Multi-purpose crops as maize, rice, soybean, and wheat are key in the debate concerning food, land, water and energy security and sustainability. While strong evidence exists on the effects of climate variability on the production of these crops, so far multifaceted attributes of droughts—magnitude, frequency, duration, and timing—have been tackled mainly separately, for a limited part of the cropping season, or over small regions. Here, a more comprehensive assessment is provided on how droughts with their complex patterns—given by their compound attributes—are consistently related to negative impacts on crop yield on a global scale. Magnitude and frequency of both climate and yield variability are jointly analysed from 1981 to 2016 considering multiscale droughts, i.e., dry conditions occurring with different durations and timings along the whole farming season, through two analogous and standardized indicators enabling comparison among crops, countries, and years. Mainly winter wheat and then spring wheat, soybean and the main maize’s season reveal high susceptibility of yield under more complex drought patterns than previously assessed. The second maize’s season and rice present less marked and more uncertain results, respectively. Overall, southern and eastern Europe, the Americas and sub-Saharan Africa presents multi-crop susceptibility, with eastern Europe, Middle East and Central Asia appearing critical regions for the most vulnerable crop, which is wheat. Finally, yield losses for wheat and soybean clearly worsen when moving from moderate to extreme multiscale droughts.

## Introduction

Maize, rice, soybean, and wheat represent a significant share of the world’s agricultural production, and they are crucial to achieve both food and energy security^1,2^. The human consumption vs. animal feed competition nourished the debate about the environmental footprints due to land and water exploitation and to greenhouse gas emissions for growing crops^3,4^, and about synergies between sustainable and healthy diets^5,6^. Moreover, the same crops represent biomass-energy sources for biodiesel (maize, rice, wheat) and bioethanol (soybean)^7^, key under mitigation targets from the Paris Agreement^8^ and currently sharing 13% of global croplands^6^. The Paris Agreement also paid attention to adaptation, so that food and energy sustainability and security should be analysed not only under past conditions but considering a likely range of future climate outlooks^9,10^.

Better quantifying the climate effects on the yield of global major crops from historical data is propaedeutic to any estimate of future climate impacts on agriculture and its connected sectors and systems (food, energy). There is consolidated knowledge about associations between climate and crop yield anomalies^11–23^, in some cases projected along future time horizons^24–28^, adopting different approaches and climate predictors, taken either as primary variables^11–15,18–25^ or after combination into indices^16,17,26–28^. All these efforts are valuable but remain fragmented as they consider one or two drought attributes, only part of the cropping season, or they focus on limited regions.

Better understanding the role of compound drought attributes—magnitude, frequency, duration, and timing—is thus crucial before any projection the likely impacts of expected climate variability on crop yields, guiding countries in proper decisions and investments for agriculture under long-term strategic planning.

In this work, we complement the existing knowledge by considering complex patterns of droughts through their compound attributes. We consider the reference period 1981–2016, adopting the well-known Standardized Precipitation Evapotranspiration Index (SPEI)^29^ to assess drought occurrence and formulating an analogous new index on interannual yield anomalies, the Standardized Yield Index, SYI. The SPEI is fed by monthly precipitation and potential evapotranspiration balance, and it represents better than other precipitation-based drought indicators the potential out-of-normal soil moisture conditions (excess or deficit), for which measured data are not so dense as for climate data. The suitability of SPEI to be used as proxy of soil moisture, including different layers, was assessed by several studies over different world’s regions^30–34^ as well as is supported by the latest (sixth) Assessment Report of the Intergovernmental Panel on Climate Change (IPCC)^35^, which preferred SPEI over other indices also due to its sensitivity to evapotranspiration and its multi-scale and classification characteristics allowing to study the different drought attributes. In this work, the SPEI has been computed for different durations (one or more months) and timings along the farming season (encompassing pre-sowing conditions). Short to long duration SPEI can be considered closely related to the shallow and deep layer soil moisture dynamics, respectively, and are useful to include the effects of fast to slow drought variability^27^. The SYI is expressed in the same way than SPEI, but it is fed by yearly yield values thus referring to unique duration and timing (representative of the farming season and its end, respectively). The SYI was calculated using the yields values for the different cropping systems (i.e., seasons of farming) for the four major global crops mentioned before. Being standardized indices, SPEI and SYI allow looking at moisture and yield anomalies with respect to the considered reference period, focusing on interannual variability and after excluding the effect of long-term dynamics like global warming, CO_2_ increase, fertilizer inputs, know-how and technological improvements in farming practices. Also, countries are better comparable one another regardless of their typical climate patterns and/or yield regimes. Moreover, SPEI and SYI have been classified into equivalent magnitude levels or classes (from normal conditions to extreme anomalies) that have been analysed, in terms of co-occurrence frequency, for the different SPEI durations and timings. Building on such a categorical representation (i.e., into classes) of yield and moisture deviations, any significant association was searched between anomalous (either lower- or higher-than-normal) both moisture and yield. A contingency approach, to our knowledge never adopted in studies on moisture-yield relationships, was applied to first count the cases (frequency) of combined either analogous or opposite moisture and yield anomaly, exploiting macro-classification (lower-than-normal vs. higher-than-normal) of moisture and yield deviations. Second, the attention was paid more on the attribute of drought and yield magnitude, exploring if lower-than-normal yields have been significantly associated to lower-than-normal moisture conditions with respect to normal or wetter-than-normal conditions, as well as if lower-than-normal moisture conditions were significantly more concentrated in years having registered lower-than-normal yields. Finally, the co-occurrences of drought and lower-than-normal yield were examined more in detail focusing on their moderate, severe and extreme classes of magnitude.

## Results

The four crops and their cropping systems analysed reveal strong association between obtained yield and climate variability observed during the farming period. First, the role of *duration* and *timing* of anomalous (i.e. different than normal) moisture conditions on the *frequency* and *magnitude* of co-occurring anomalous moisture and yield is analysed considering SPEI and SYI macro-classes, e.g. lower yields under drier conditions (*LY_D*), higher yields under wetter conditions (*HY_W*), higher yields under drier conditions (*HY_D*) and lower yields under wetter conditions (*LY_W*). Then, within the macro-class *LY_D*, focus is given to single anomalous classes of magnitude (moderate, severe, extreme) of SPEI and SYI and on their co-occurrence frequency.

### Anomalous yield and water stress

**Figure 1:**
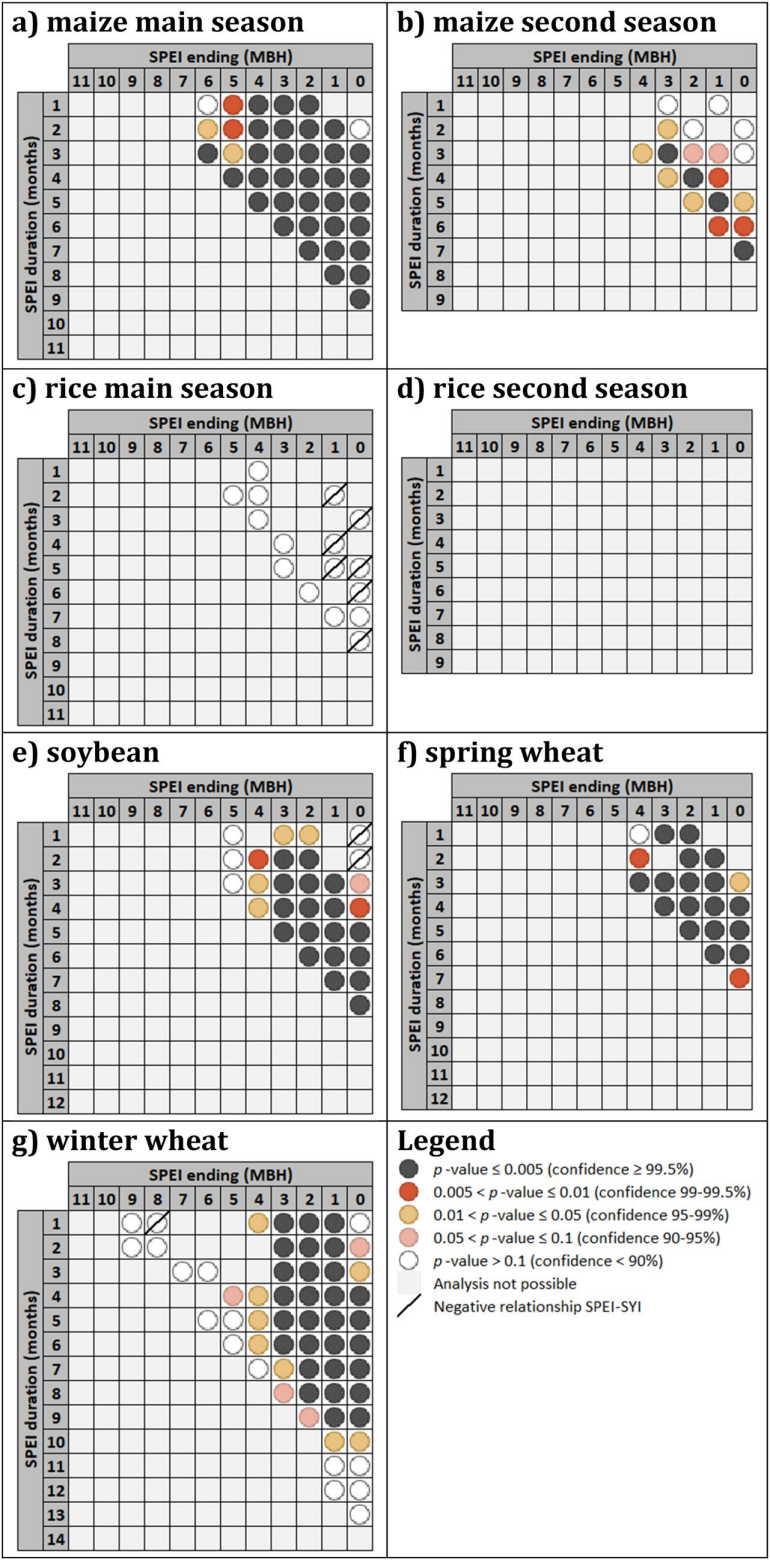
Magnitude and frequency for co-occurring lower-than-normal both moisture and yield. SPEI duration-timing matrix of merged *p*-values measuring the overall aggregated significance among: (i) Asymmetry of contingency-tables fully skewed towards the co-occurrence of lower-than-normal both yield and moisture (*LY_D*); (ii) differences in SYI magnitudes under SPEI ≤ − 1 vs. SPEI > − 1; and iii) differences in SPEI magnitudes under SYI ≤ − 1 vs. SYI > − 1. Colors refer to *p*-value classification as reported in the legend. Lower *p*-values represent more significant asymmetry towards *LY_D* (i), significantly lower SYI values under drought vs. non-drought conditions (ii) and significantly lower SPEI values identified in cases of lower-than-normal vs. normal or higher-than-normal yields (iii). Grey cells represent no minimum sample reached to do either SPEI or SYI magnitude analyses or no asymmetry fully skewed toward *LY_D*. Diagonal lines indicate the case of overall higher SYI (or SPEI) under lower-than-normal moisture (or yield). SPEI ending (in Months before the Harvesting month, MBH) represents the timing and indicates the final month of the consecutive months period for SPEI durations longer than 1 month.

Figure 1 summarizes results from in-depth statistical analysis of SPEI duration-timing contingency tables as described in “Methods”. The aggregated statistical significance among three tested assumptions is shown: (i) higher frequency of occurrence for *LY_D* against any other macro-class (referred to as “Asymmetry > 50%”, see “Methods”) (Supplementary Fig. S1); (ii) differences in SYI magnitude when SPEI ≤ − 1 with respect to when SPEI > − 1 (Supplementary Fig. S2); and (iii) differences in SPEI magnitude when SYI ≤ − 1 with respect to when SYI > − 1 (Supplementary Fig. S3). Supplementary Figure S4 is analogous to Fig. S1 but in case of asymmetry completely skewed towards *HY_W*, to capture potential associations between wet conditions and higher yields. Figure 1 is accompanied by Supplementary Table S1 presenting, besides global season lengths’ statistics for the different cropping systems, aggregated results on the asymmetry, quantifying the average prevalence of the *LY_D* macro-class occurrence with respect to any other macro-class (i.e., when the prevalence is always above 50%; see “Methods”).

By analysing SPEI duration-timing contingency tables for the main season of maize, Fig. 1a reveals that an interval extending up to 8 months before the harvesting month seems of interest for the connection between lower-than-normal yield and medium to long duration (more than 2 months) lower-than-normal moisture, with confidence at least 99.5%, confirming the importance of pre-sowing moisture regimes (looking at average season’s duration in Table S1). Shorter duration droughts (less than 3 months) confirm a very strong influence before the last couple of season’s months and, even when occurring far (more than 4 months) before the harvesting month, reveal however a considerable impact (confidence at least 95%).

The second season of maize is less represented across the World’s countries and concentrated in the subtropical areas of America and Africa. From Fig. 1b it is evident how the effects of droughts longer than 2 months extend up to 5 and 6 months before the harvesting month at least at the 99% and 90% confidence level, respectively, again entering the pre-sowing period. Interestingly, 95% confidence was found also in the case of one-month anomalous moisture happening four months before the harvesting month but with asymmetry toward *HY_W* macro-class (Fig. S4b), suggesting that short wetter-than-normal conditions around or soon after planting could significantly benefit yields.

For the main season of rice, only in case of SPEI-03 ending four months before the harvesting month (so during the initial phases of the cropping season), a correspondence between drier-than-normal conditions and lower-than-normal yields in terms of frequency is found with 90% confidence (Fig. S1c). Looking at magnitude level, drier-than-normal both one- and two-month period (SPEI-01 and SPEI-02), one month before harvesting month and at harvesting month, respectively, seem consistently leading to higher SYI if compared to when SPEI > − 1 (confidence level at least 95%), while lower SYI appears associated to drier-than-normal periods irregularly along the other durations and timings analysed (confidence between 90 and 99.5%) (Fig. S2c). Overall, lower-than-normal yields seem associated with 1-month drier conditions just before or at the very beginning of the cultivation season (confidence level between 95 and 99%, Fig. S3c). All that above prevents evincing either a clear or a significant behaviour for rice at global level (Fig. 1c).

For the rice’s second season, concentrated in south-east Asia and secondarily in central America and central Africa, asymmetry towards *LY_D* is never met, preventing any integrated analysis through Fig. 1d. Interestingly, lower-than-normal yields seem related to higher very short-duration SPEI (thus to less dry conditions) at the beginning of the cultivation season (Fig. S3d, confidence between 90 and 99%), while a 3-month wetter-than-normal period, roughly covering the first half of the growing season, seems associated to higher-than-normal yields (Fig. S4d, confidence levels between 90 and 95%).

Concerning soybean, Fig. 1e shows that for eight months in total before the harvesting date, so including at least a couple of months before planting, long droughts (lasting 5–8 months) lead to lower-than-normal yields (confidence ≥ 99.5%), while medium duration droughts (from 3 to 4 months) have the highest significance of negative impacts if occurring around planting (or slightly earlier) up to at least 1 month before harvesting month. Shorter (from 1 to 2 months) droughts have impacts when happening around the season’s mid (confidence ≥ 95%), while very short wetter-than-normal conditions slightly before this middle period seem favouring yields (confidence ≥ 99.5%, Fig. S4e). Such significant positive relationship between yield and moisture anomalies are well reflected in all the analysis components, with only short drier conditions around harvesting date or just before the harvesting month not allowing to evince significant or strong effects.

Looking at spring wheat, Fig. 1f shows how the medium-long term anomalous dry conditions (lasting 3 to 7 months) negatively affect yields when encompassing stages before to soon after planting until harvesting (confidence ≥ 99.5%). Drought periods with shorter durations (1–2 months) have impacts if occurring around or soon after the mid of the growing season (confidence ≥ 99.5%), while a couple of wetter-than-normal months just before this middle period can favour yields (Fig. S4f). No significant effect is found for very short (1-month) anomalous moisture regimes close to the harvesting date (Figs. S1f, S2f, S3f), except one month before harvesting, revealing lower SYI for drier-than-normal conditions and more frequent higher-than-normal yields under wetter-than-normal conditions (confidence 95–99%, Figs. S2f and S4f, respectively).

For winter wheat, a clear signal (confidence ≥ 99.5%) is that strong negative effects on yields arise from very short to half year duration droughts if occurring or protracted at least until after the middle of the growing season (i.e., 3 months before harvesting month), or from exceptionally long droughts (7 to 8 months) if ending in the last season’s quarter (Fig. 1g). Good confidence is found (90–99%; Fig. 1g) for the negative impacts on yields from short-lasting drier-than-normal periods (2 to 3 months) when ending at harvesting, as well as from very short and medium-lasting anomalous periods (1 month and 4 to 6 months, respectively) ending before the growing season’s mid, when instead benefits on yields due to short (2–3 months) wetter-than-normal conditions are detected (Fig. S4g).

### Country and year susceptibility

**Figure 2:**
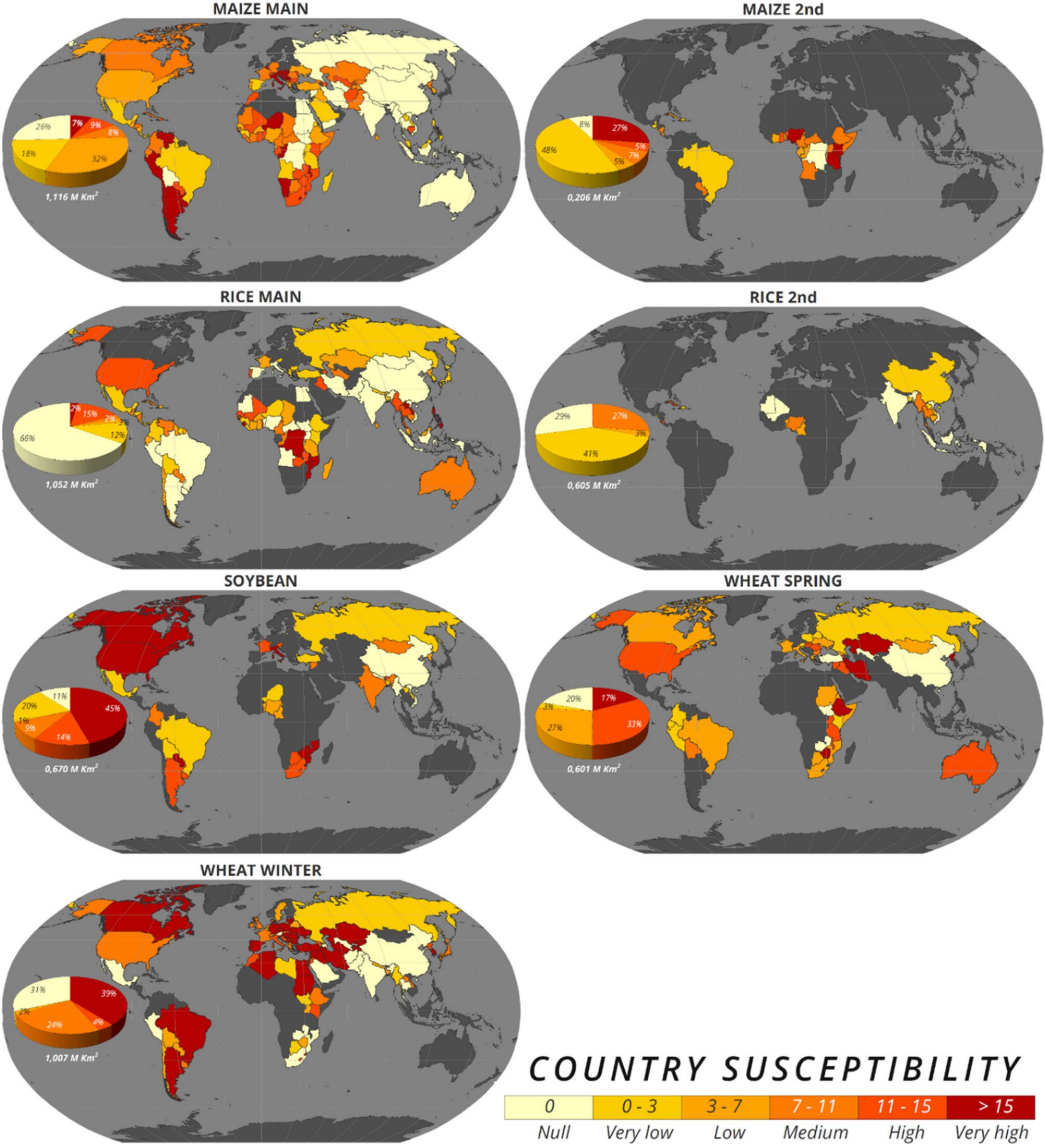
Country susceptibility to drought-low yield association. Average occurrence, for each country, of SPEI duration-timing combinations with SPEI ≤ − 1 across all the years with SYI ≤ − 1. The values obtained from all cropping systems are classified into susceptibility classes considering five quantiles while zero is presented separately (light yellow) and distinguished from “no data” land areas (dark grey). Pie diagram represents the susceptibility classes’ share (within cultivated surface) among the coloured countries (i.e., excluding no data areas); the global cultivated area is reported below the pie in Km^2^.

To highlight the world’s countries where the farming season seems more susceptible to droughts occurring in a rather complex patterns (e.g., in different growing phases and/or with different lengths), Fig. 2 summarizes the mean counts of SPEI timing and duration combinations in case of drought conditions (SPEI ≤ − 1) for any annual occurrence of SYI ≤ − 1 (lower-than-normal yield). The considered countries are listed in Supplementary Table S2, while the cultivated surface per country and cropping system is reported in Supplementary Table S3, and the within country spatial distribution of crops is shown in Fig. S5. For the main season of maize, especially the western side of the globe contributes to the classes with medium to very high susceptibility (i.e. more than 7 combinations on average for any anomalous yield occurrence) occupying around 268 000 km^2^ (24%) of lands where the crop is grown, with hotspots (high to very high susceptibility, i.e., more than 11 combinations) in western South America, Sub-Saharan Africa, and southern-eastern Europe. Most susceptible countries for the main rice season are concentrated in sub-Saharan Africa, South-Southeast Asia, and secondarily in the U.S. and Australia, with most concerned lands (medium to very high susceptibility) covering around 208,000 km^2^ (20%) of cultivated surface. For the second season of maize and rice, extending mostly within the tropical belt, countries more suffering from complex droughts are concentrated in Central Africa and in Central American Islands, respectively, and both revealing much less than 200,000 km^2^ (however more than one third and one fourth, respectively) of cultivated lands from moderately to very highly impacted. The most affected countries for soybean cultivation are in North America, in the extreme either south America or south Africa and in southern Europe, with moderately to very highly susceptible lands occupying around 460,000 km^2^, more than two thirds of territories growing the crop, and the highest class alone covering almost half (45%) of these territories. Spring wheat suffers from complex drought patterns especially in Central Asia, in the easternmost side of central and southern Africa, in U.S. and Australia, covering 50% of the total 600,000 km^2^ cultivated with this crop. Winter wheat yield appears from moderately to very highly exposed especially across the trans-Mediterranean region surrounded by North Africa, Middle East, central Asia, and many European countries extending from southern to central-eastern Europe, covering around two thirds of the total cultivated surface (1 Mil km^2^). Among nations hosting both wheat cropping systems, the conditions of Central Asia, Middle East and Eastern Europe seem particularly problematic. Looking at all crops together, the American continent, sub-Saharan Africa and both southern and eastern Europe reveal the highest vulnerability.

**Figure 3:**
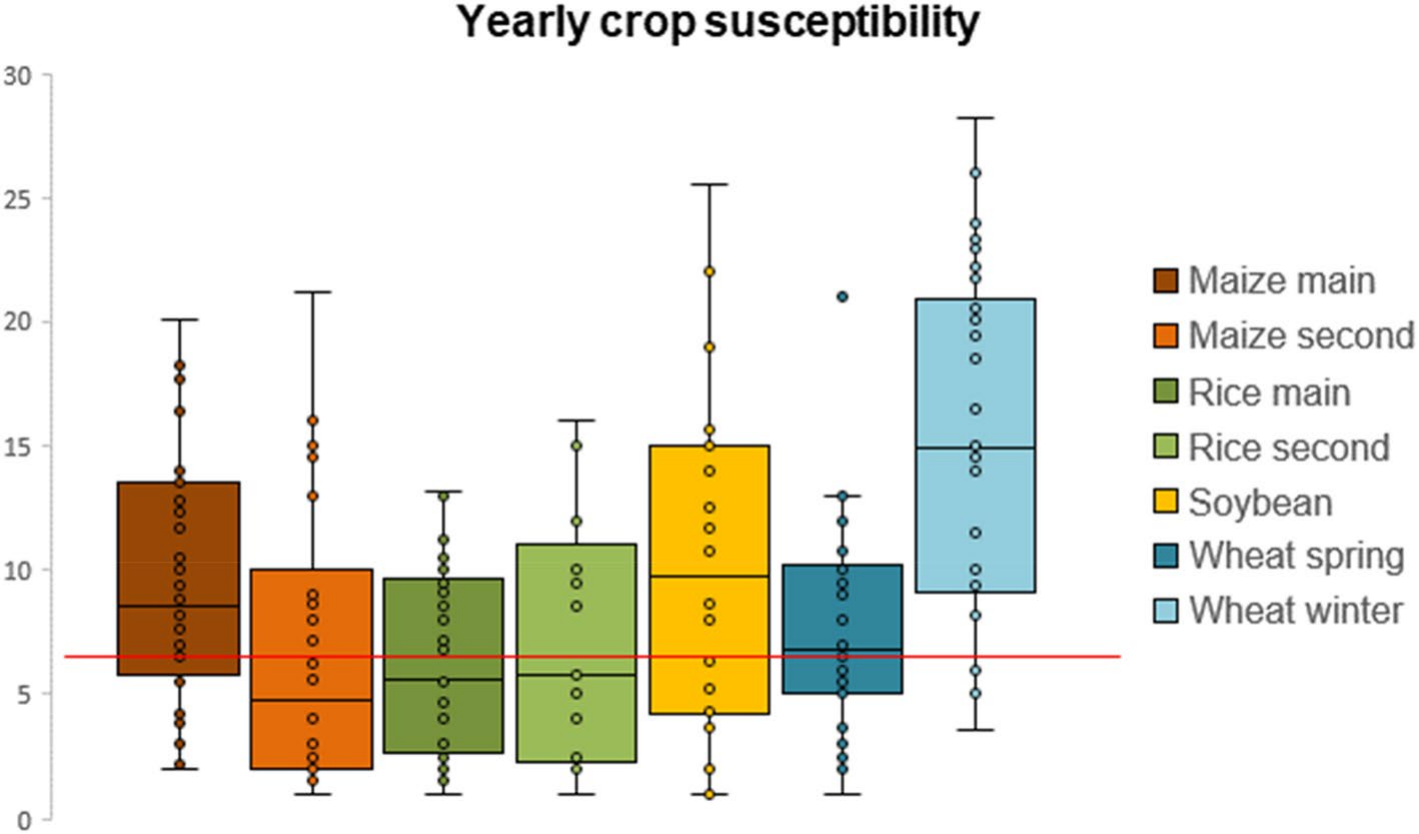
Yearly crop susceptibility to drought-low yield association. Average global occurrence, for each year and cropping system, of SPEI duration-timing combinations with SPEI ≤ − 1 across all the occurrences of SYI ≤ − 1. Single points (some of which overlapping) represent each one year, the box limits indicate the 25th and 75th percentile while the internal horizontal line is the median. The whiskers represent the minimum and maximum after exclusion of outliers (i.e., values outside 1.5 times the interquartile range from the 25th and 75th percentile, respectively). Red line is the 2nd quantile among occurrence values assumed, as in Fig. 2, as the limit between *Low* and *Medium* susceptibility.

Figure 3 shows, for each cropping system, the distribution of the average annual counts of SPEI timing and duration combinations in case of droughts (SPEI ≤ − 1) for any occurrence of SYI ≤ − 1, at global level. It is clear how winter wheat reveals most frequently (i.e., well above the limit between low and medium susceptibility as classified for Fig. 2) complex drought arrangements along the farming season matching with anomalous lower-than-normal yields, followed by soybean and the main maize’s season, and secondarily by spring wheat. Rice, and maize in its second season, presents the lowest combinations’ occurrences. Supplementary Table S4 details the split of such a susceptibility per crop system, country, and year, highlighting 1983/1984, 1992, 2000 significantly more susceptible than others, confirmed by crop aggregated analysis in Supplementary Table S5, and secondarily years 1989, 2003, 2008, 2011 and 2015.

### Drought and lower yield co-occurrence

Table 1 summarizes how, within the *LY_D* macro-class, the co-occurrence of SYI magnitude classes equivalent or severer than SPEI classes prevails against the sum of all the co-occurrence between SYI and SPEI classes (i.e., dominance occurs when the share is above the threshold of 66.7%; see “Methods”). The share of co-occurrence of SYI magnitude classes severer than SPEI classes is also estimated, considering 50% as threshold to assume the dominance with respect to the total co-occurrences of not analogous classes (see “Methods”). For the former, the dominance is evinced for all cropping systems, being 77% as weighted average function of contingency tables analysed for each cropping system and, except for the second maize season, dominance above the threshold is found for more than two thirds of the duration-timing contingency tables analysed (87% as weighted average). For the latter, the dominance is found, although weaker, for the majority of cropping systems, being 53% as weighted average and above threshold for a little less than two thirds (i.e., 64% as weighted average) of contingency tables analysed, exceeding this share for soybean (94%) and winter wheat (72%) more reliably than for the second rice season (100%), as the first two crops can rely on more contingency tables than the third one.

**Table 1:** Aggregated results, per cropping system, in terms of average dominance—across contingency tables analysed (second column)—in the co-occurrence of SYI magnitude classes equal or severer (third column), or only severer (fourth column), than SPEI classes within the lower-than-normal both moisture and yield macro-class (*LY_D*).

Cropping system	N. of contingency tables analysed	% of co-occurrence where SYI class ≥ SPEI class [and % of contingency tables with co-occurrence > 66.7% among all classes]	% of co-occurrence where SYI class > SPEI class [and % contingency tables with co-occurrence > 50% among non-analogous classes]
Maize main	42	77% [95%]	51% [52%]
Maize 2nd season	25	71% [60%]	46% [44%]
Rice main season	33	74% [73%]	46% [45%]
Rice 2nd season	12	84% [92%]	68% [100%]
Soybean	33	82% [97%]	64% [94%]
Wheat (spring)	25	76% [80%]	52% [48%]
Wheat (winter)	85	78% [95%]	54% [72%]
Average		77% [87%]	53% [64%]

Enter brackets, the share of duration-timing contingency tables presenting above threshold dominance is reported as calculated over the total number of contingency tables analysed.

**Figure 4:**
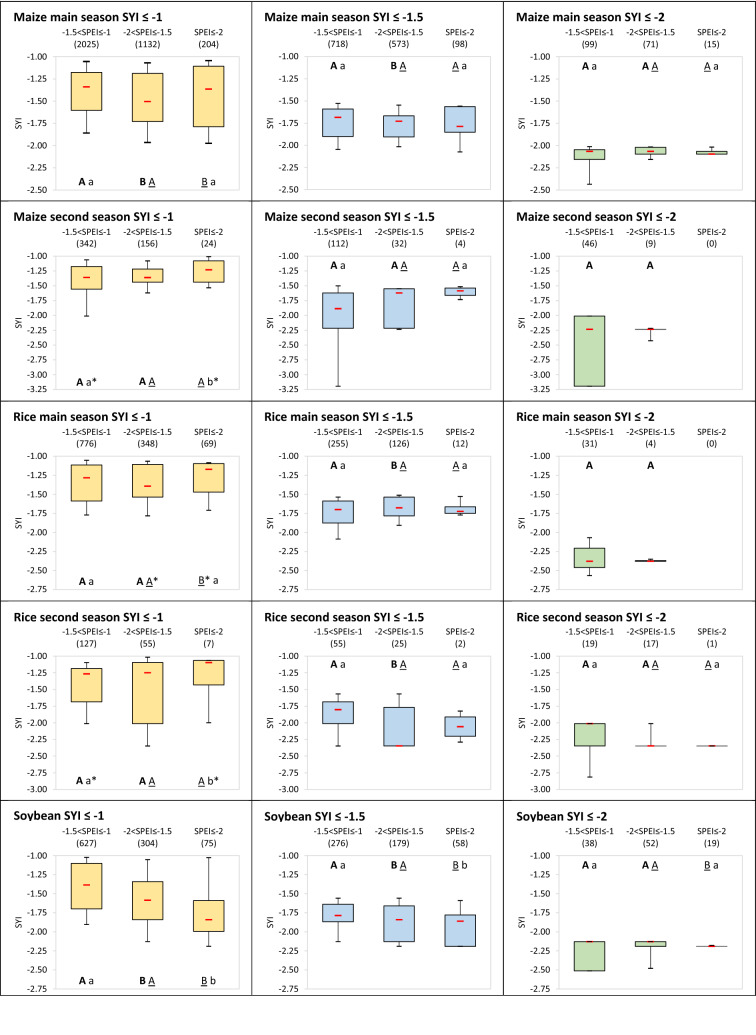

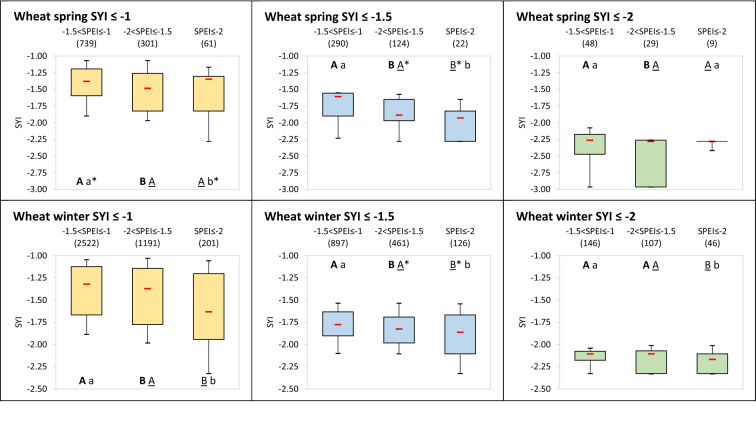
SYI values under drought related SPEI classes. Distribution of SYI values when SYI ≤ − 1 (left panel, yellow box plots), SYI ≤ − 1.5 (central panel, blue box plots) and SYI ≤ − 2 (right panel, green box plots) for − 1.5 < SPEI ≤ − 1, − 2 < SPEI ≤ − 1.5 and SPEI ≤ − 2. The box boundaries indicate the 25th and 75th percentile, the whiskers the 10th and 90th percentiles, and the red bar the median. Numbers enter parentheses indicate the sample available under each SPEI class and SYI range, across the years, countries and SPEI timings and durations considered. The letters with the same style (bold capital, underlined capital, or lowercase) indicate (if different) that in the pair-wise comparison between SPEI classes the related SYI values are significantly different at the 95% confidence level (*p*-value ≤ 0.05), the asterisk meaning instead a confidence level between 90 and 95% (0.05 < *p*-value ≤ 0.1).

Figure 4 focuses on the distribution of anomaly magnitude for ranges of lower-than-normal yields (SYI ≤ − 1; SYI ≤ − 1.5; SYI ≤ − 2) across different classes of SPEI, showing how there is high significance for winter wheat and soybean for which the severer is the moisture deviation from normal conditions the higher is the negative anomaly of yields. Confidence is lost only in case of extreme lower-than-normal yield (SYI ≤ − 2) between close classes of moderate and severe drier-than-normal conditions for winter wheat, and between the moderate class and either the severe or the extreme one for soybean, the latter however represented by a limited sample. The spring wheat follows in results’ significance, with cases of SYI ≤ − 1 losing confidence in the difference between severe and extreme SPEI classes, while those under SYI ≤ − 2 showing significance only in the divergences between moderate and severe SPEI classes. For maize in its main season, some confidence is found only for SYI ≤ − 1 and SYI ≤ − 1.5 in the difference between moderate and severe SPEI classes, while the extreme class presents no significant differences from the others or, in one case under SYI ≤ − 1, an opposite behaviour, i.e., significant higher SYI for the SPEI extreme drought class with respect to the severe one.

For rice in its main season, as well as for the second season of both maize and rice, contrasting results are found, with few significant cases and sometimes with overall higher SYI under lower SPEI classes; the general low confidence can be also due to less populated samples for these cropping systems.

## Discussion and conclusions

We considered duration, timing, frequency, and magnitude of lower-than-normal moisture conditions to investigate the possible impact of droughts, under more complex patterns than previously investigated, on the productivity of world’s key crops. We exploited the Standardized Precipitation Evapotranspiration Index (SPEI) and an analogous indicator of yield interannual variability (Standardized Yield Index, SYI) enabling robust comparison among cropping systems, countries, and years.

Our consideration of drought at multiple timescales (durations and timings) during the farming season (encompassing pre-sowing until harvesting) complements the usual practice of considering, for global scale evaluations, either the SPEI or other climate indicators only for a few, short and/or fixed periods (e.g. the growing season, several months before harvesting—the reproductive period—or 1 year)^13,14,16,17,26^, while neglecting sowing antecedent moisture conditions which are also important for the soil workability and crop development^36,37^.

The strong relationships among multiscale SPEI and crop yield was already observed in some global to regional studies^38–40^. In particular, the association between moisture and yield anomaly variability was highlighted at (sub)regional scale using SPEI and the standardized yield residual series (SYRS) (^41^), e.g. for Eastern Europe^41–43^, Spain^44^, North America^45,46^ and China^47–49^. While these works have mainly explored the correlation between moisture and yield anomaly magnitudes using different regression models, focusing on crop- and site-specific calendars for the considered regions and thus reducing the drought durations and timings analysed, we provide here a wider analysis integrating drought magnitude, frequency, duration, and timing under a global view with national-level detail mostly driven by the spatial granularity of crop calendars. Moreover, our approach differs from those that combined the SPEI and the SYRS due to the identical detrending, standardization and classification procedure we applied to the SPEI and the adopted SYI.

We show how the yield of the main crops analysed is susceptible to more complicated drought patterns than evidenced by previous valuable studies for specific crops and regions, and that such a complex drought-low yield connection is a consistent feature at global scale. In particular, we reinforce earlier findings about the high vulnerability to drought of wheat, soybean, and maize, as well as former conclusions about no substantial or clear impacts on rice, for which the temperature regime seems the most important driver and whose water demand is mostly covered through irrigation^11,14,16,22,38,47^. The shorter SPEI scale (up to 3–4 months) is confirmed a good indicator of yield loss as formerly found for maize^43,48,50,51^, soybean^51^, and spring and winter wheat^43,46,52,53^. Moreover, we strengthen the previous scattered findings on how medium- to long-term droughts (lasting more than 6 months) have a noteworthy influence on crop yield, although their association with lower-than-normal yield is significant mainly when such extended droughts protract until the second part of the growing season. Indeed, on one side, the reproductive and grain development stages are more sensitive than early vegetative phases to shorter term low moisture conditions as shown for North America^38,46^, China^40,48,49^ and Iberia^44,52^. On the other side, the crop yields also pay for the accumulation of water deficit due to longer scale droughts encompassing previous seeding emergence and vegetative stages, especially for wheat^44,46,48,49,52,54^ but also for maize^54^. For maize and wheat, earlier and later stages in particular were already found vulnerable to short- and long-term droughts, respectively (^41,42^). Long-lasting drought encompassing the winter wheat growing season plus 90 days (around 3 months) before sowing was found highly correlated with negative yield anomalies^55^. In case of very short duration and severe droughts at nearly weekly scale it was assessed that, if occurring during the seeding period, they negatively affect winter wheat production^56^. All that confirms the key role of moisture conditions at (pre)sowing stage as we also highlighted, although the sub-monthly time scale was not explored in our study. We also found that the severer the drought, the severer—or even amplified—the yield anomaly; this matches with some previous findings that, in general, droughts of higher magnitude have greater impacts on winter wheat yield during critical growth stages^56^.

We reveal concurrent susceptibility for key crops across years and countries. Some of the top 10 producing nations (according to FAOSTAT^57^ for the period 2015–2019) appear particularly vulnerable (Fig. 2): Argentina, Canada and Romania for maize; North America, Paraguay, Argentina, and India for soybean; North America, Brazil, Argentina, Ukraine, France for wheat, and Thailand, Myanmar, and Philippines for rice, although this crop presents less clear results. All that threatens the global breadbaskets as already identified for recent decades^22,58^, which registered an increasing trend of chronically drought-prone lands^59^ and with global grain production areas presenting drying trends especially in developing countries^60^.

Our results reveal as globally susceptible to complex drought patterns, with different significance, the years 1983/1984 (maize and rice), 1989 (winter wheat), 1992 (maize, rice, and soybean), 2000 (maize), 2003 (maize and winter wheat), 2008 (wheat), and 2011 and 2015 (rice). Interestingly, these years were interested by weak to strong El Niño (1992, 2003 and 2015) and strong La Niña (2000) and by consecutive El Niño and La Niña (1983/1984, 1989, 2008, 2011), already recognized affecting yield in some world areas, in different ways function of the crop considered^13^. In particular, our work consolidates these years as critical for yields especially in terms of complex drought patterns and at global level, which is important for the food system given that food consumption is not only based on local production, but international trade has strong importance^61^.

The expected drought regime for the future, in terms of simultaneous rising of the frequency, duration and magnitude of short, medium to long term droughts (^35,62–64^), will likely worsen the susceptibility as also evinced for the same crops under future climate considering different indicators and approaches^24–27^.

In particular, the latest IPCC Sixth Assessment Report (AR6)^35^ mentions a medium confidence in the observed increase of agricultural droughts in all continents, as well as high both confidence and likelihood that with global warming from 1.5 to 4 °C the land areas affected by increasing drought frequency and severity will expand, due by decreasing precipitation and increasing atmospheric evaporative demand. Concerning soil moisture drought duration, in the Mediterranean regions—which is revealed particularly critical in our study—it is expected to increase under + 1 °C to + 3 °C in a range from 41 to 125 days, representing + 46% and + 346% than in the late XX Century^35^.

Under raising food demand to nourish growing and increasingly urbanized populations^65^, future drought patterns will likely affect agricultural productivity growth^66^ and thus food security, international trade, and volatile food price and provision, potentially leading also to criticalities in terms of conflicts, livelihood insecurity and migrations^67–71^, as well as exacerbating competition for bioenergy investments under climate change mitigation targets^72,73^.

Potential limitations of this study should be addressed in future research.

First, other drought indices could be more representative for some world regions or countries. Indicators considering only precipitation are suitable for subtropical areas (^39^), although those based on soil moisture could better represent the water budget component key for crop growth. However, the possible use of soil moisture indicators suffers from several drawbacks function of the data source considered (https://climatedataguide.ucar.edu/climate-data/soil-moisture-data-sets-overview-comparison-tables). If relying on ground-based soil moisture measurements, related networks are very sparse and cover very short or discontinuous periods. If monitored through remote sensing, gaps in soil moisture coverage are due to satellite orbits and to the fact that sensors cannot see soil where vegetation is dense, leading to relevant spatial gaps. Physically based land surface models, with and without data assimilation, retrieve soil moisture using meteorological variables as input (e.g., precipitation, radiation, wind, temperature, humidity), but they are prone to systematic errors mostly due to model physics, soil and vegetation datasets used for model initialization, parameterization and/or validation. Our study is in line with the IPCC AR6 that relies on indicators based on precipitation and potential evapotranspiration (PET) for assessing agricultural (soil moisture) droughts. Indeed, while it is true that precipitation- and PET-based drought indicators, as the SPEI exploited here, could overestimate soil moisture deficit in water limited regions, they are good for (sub)humid regions (where actual evapotranspiration is not limited by soil moisture and tend to PET), and allow to also consider hydrological drought in terms of irrigation providing water bodies whose surface resource is not limited, as well as to account for crop water consumption in irrigated lands, common in semi-arid areas. Due the limits of temperature-based PET estimates, we considered here a dataset relying on the formulation based on the Penman Monteith equation, the same recommended in the IPCC AR6 (^35^).

Second, a level of inherent uncertainty is related to that of input data and related processing (see “Methods” and references therein). Climate data derive from an interpolation of station measurements, each having different lengths of available time series, also for the different variables, affecting the accuracy of monthly series provided in gridded format. The crop yield dataset is based on model estimates, and it is not free from error due to imperfect modelling, inaccurate inputs, misreporting in agricultural census statistics, and use of time-constant information; in this context, the use of a standardized indicator limits misleading conclusions sensitive to the choice of the input dataset. Another potential way to exploit yield time series is selecting alternative detrending methods, as well as different methods according to (sub)country peculiarities. Moreover, the beginning and ending years of the dataset (i.e., 1981 and 2016, respectively) have many missing values in the Southern Hemisphere because crop growing season in the region often span two calendar years and yields cannot be estimated due to this incomplete season duration; this could be one of possible reasons of not having found significance in the multi-scale drought counts after the very strong and long El Niño event spanning 2015–2016. The dataset on the fraction of harvested area has the main limit of not distinguishing crop systems and of being not temporally explicit but an average around year 2000, which could neglect changes (restrictions, expansions) in cultivation areas along the analysis period to be associated to the multi-year yield dataset. Finally, due to the global scale addressed, the country-level detail cropping systems’ calendars could affect the spatial accuracy of the analysis, in particular for very extended countries and/or less representative cropping systems. For example, while the second maize season seems regarding only some world’s regions and at the same time it constitutes a limited share of total maize yield, the second rice season represents instead a significant share of rice total yield (^16^), and even a third season for rice seems occurring in the highly producing China (^74^). Moreover, the distinction of winter and spring wheat in the crop calendar, as well as of main and second season, requires caution (^75^). To reliably reproduce possible calendars, recent efforts highlighted the need of considering, in addition to crop biological requirements for heat, chilling, and moisture, the field workability due to snow cover and heavy rainfall as well as multi-cropping patterns (^76^), as well as satellite-derived phenology products (^75^).

Third, other factors potentially impacting crop yield in the analysed period should be considered: on the short-term, disturbances like pests or pathogens, although proper assessment is prevented by lack of data (^22^); on the long term, crop variety, ozone exposure (^77^) or CO_2_ fertilization effects deserve attention, although elevated CO_2_ seems offering no benefit for winter wheat during drought years in Europe (^78^). Last, our study does not account for irrigation practice and its interaction with drought; this depends on the lack of global data about actual irrigation amount, although useful information exists on equipped area (^79^), and on the consideration that the contribution of irrigation to yield increment remains uncertain and it is also function of performances and/or continuous investments related to hydraulic engineering for water diversion and cross-basin water transfer (^80^).

Overall, our comprehensive analysis could add valuable information to deal, in a harmonized way, with drought impacts on the global agricultural production for multipurpose (food and energy security) crops. The recognition of more complex drought patterns associated with global yield losses can help to target the deployment of early warning and decision support systems under short-term predictions and long-term projections of climate regime, e.g. to identify lower yield probability based on future drought patterns using identification and sampling approaches as done for other impacts^81^ or embedding drought indicators as modifiers in crop simulation models^82^. In turn, the approach used in our study can contribute to supporting agriculture development and related management options in the context of climate mitigation and adaptation policies, like fine-tuning crop calendars or implementing other measures as alternative cultivars, additional irrigation, and crop migration^83–85^, while also guiding governments, businesses and international institutions toward more effective contingency plans, crop reserve management and/or trade strategies^22^.

## Methods

### Datasets and data analysis

All the input datasets used in the study have been elaborated to enable consistent comparison across countries and along years from 1981 to 2016, as detailed in the paragraphs below.

### Crop cover and spatial weighting strategy

The extent of lands interested by cultivation of the four major crops analysed (maize, rice, soybean, wheat) has been extracted from the dataset “*Harvested Area and Yield for 175 Crops year 2000*”^86^, originally available on a regular grid spacing of 5’ (≈ 0.0833°). Such data represent the average fractional proportion of a grid cell that was harvested for the considered crop during the 1997–2003 period (circa year 2000). Based on the final country scale of the analysis (see also next paragraphs), these datasets were first resampled by keeping, for each new coarser resolution (0.5°) pixel, the average value among the 36 (6 × 6) finer resolution (≈ 0.0833°) pixels therein (*FC*, Eq. 1).

1 $${FC}_{c,j}=\frac{1}{36}{\sum }_{i=1}^{36}F{C}_{c,i}$$

where *FC* stands for fraction cover and the subscript *c* refers alternatively to maize, rice, soybean, or wheat, based on the crop considered, while *i* represents each of the 36 finer resolution pixels belonging to the new coarse resolution pixel *j*.

Successively, these average *FC* were summed within each country (*Sum_FC*, Eq. 2) and this value was used to extract a weight (*W_FC*, Eq. 3) to be then applied to the gridded values of the considered climate variability indicator (see later) to obtain a country-average of it.

2 $$Sum\_F{C}_{c,cnt}={\sum }_{j=1}^{n}{FC}_{c,j}$$

3 $$W\_F{C}_{c, j}=\frac{{FC}_{c,j}}{{Sum\_FC}_{c,cnt}}$$

where *n* is the number of coarser resolution (0.5°) pixels *j* in the country *cnt*. The map of the countries was derived from the Global Administrative Areas Database (GADM; https://gadm.org/) and converted into a 0.5° × 0.5° grid assigning an ID to each country (see Table S2 in Supplementary Information for the countries’ ID).

### Crop calendar and spatial aggregation

The planting and harvesting dates for each crop considered have been accessed through the *Crop Calendar Dataset*^75^, available at both 5’ and 0.5° grid spacing. The latter resolution, derived from the re-gridding of the former, has been considered here due to the spatial resolution of the analysis driven by climate and yield data (see next paragraphs). Calendar dataset refers to seven different cropping systems for the four crops analysed: main and second season for both maize and rice, winter and spring sown for wheat, and only one system for soybean.

Among the *Crop Calendar Dataset* parameters, both planting and harvesting start, end and average dates are reported expressed as Julian day of the year, while planting and harvesting intervals, as well as the total crop season length, are reported as total number of days. The average dates for planting and harvesting have been considered here and converted into a value from 1 to 12 corresponding to the calendar month the dates belong to; the month-based values have been then averaged (keeping the nearest integer value) over the country, so to have a single representative planting and harvesting month per country.

### Climate variability indicator and input datasets

The climate variability indicator adopted is the Standardized Precipitation Evapotranspiration Index (SPEI^29^), used in general to quantify the deviations from normal potential soil moisture conditions (deficit or surplus) by combining thermal and humidity regimes. This index is similar to the well-known Standardized Precipitation Index (SPI^87^) but it merges monthly series of precipitation (P, mm) and potential evapotranspiration (PET, mm) rather than using P alone, and thus it indirectly considers temperature and other variables, depending on how PET is calculated. While SPI is a multi-scalar indicator suitable for meteorological drought assessments, SPEI is valuable to better represent combined air, soil, and vegetation processes and thus four different attributes of agricultural droughts. First, the *duration*: the balance precipitation minus evapotranspiration (P-PET) at the basis of SPEI can be calculated for one or several consecutive months. Second, the *magnitude*: given a climatological reference period, the SPEI quantifies for each month or consecutive months in the period how much the potential soil moisture (P-PET) deviates from the normal, standardizing the values for each month and location using log-logistic distributions and classifying them as normally, moderately, severely, or extremely dry/wet (Table 2). Third, the *timing*: the duration can be assumed starting (or ending) in any month of the year, representing drought onset (or cessation). Finally, the *frequency*: over the climatological period, the occurrence of each magnitude class, for a given duration considered and for any onset (or cessation) can be calculated.

**Table 2:** SPEI classification into classes and macro classes.

Value classification	Class description	Macro class
SPEI ≤ − 2	Extremely Dry (ED)	Dry (D)
− 2.0 < SPEI ≤ − 1.5	Severely Dry (SD)	
− 1.5 < SPEI ≤ − 1.0	Moderately Dry (MD)	
− 1.0 < SPEI < 1.0	Normally Dry to Wet (N)	Normal (N)
1.0 ≤ SPEI < 1.5	Moderately Wet (MW)	Wet (W)
1.5 ≤ SPEI < 2.0	Severely Wet (SW)	
SPEI ≥ 2.0	Extremely Wet (EW)	

For the purpose of the present work, the high-resolution Climate Research Unit Time Series (CRU-TS) dataset version 4.03 was exploited^88^. CRU-TS4.03 is available from 1901 to 2018 in monthly series at 0.5° grid spacing and, among variables, P and PET are provided, the latter calculated according to the Penman Monteith formula^89^. From these data, years from 1981 to 2016 were extracted to match the availability of yield data (see below paragraph). The SPEI was calculated using the SPEIbase R-package (https://zenodo.org/record/834462#.X8Zq381KhPY) and it differs from the database currently available from the same authors under the same CRU dataset (https://spei.csic.es/database.html) as in the present work the reference period is made of 36 years (1981–2016) rather than 118 (1901–2018) and the P-PET has been here linearly detrended to disentangle the global warming effect on evapotranspiration and concentrate rather on interannual variability of precipitation and other meteorological variables^14,16–18,20,21,23^. Detrending was applied directly on the monthly P-PET time series, and not on the single P and PET time series to avoid losing physical consistency between the two variables.

The SPEI was calculated for each land cell of the CRU-TS4.03 grid with values different than no data (i.e., except Antarctica) along the 36 years. All the possible ending months *m* within a year (1 to 12, called hereafter *timing*) have been considered and each SPEI has been dated according to the ending month of the duration considered (e.g., the 3-month SPEI—SPEI-03—covering months from January to March, is dated as March). Then, given the maximum length of 12 months found for crop seasons among the cropping systems and across the world (i.e., winter wheat for central-western Russia), and aiming at considering the soil moisture also before the planting period to take into account potential anomalous climate conditions during soil working operations, SPEI was calculated for durations *d* from 1 to 14 consecutive months along the period 1981–2016.

Then, for each crop *c*, SPEI values have been averaged at country level weighting each cell in the country *cnt* (Eq. 4) according to its actual crop fraction cover (see Eq. 3, also for the explanation of subscripts).

4 $${SPEI}_{c,cnt,d,m}={\sum }_{j=1}^{n}{SPEI}_{j,d,m}*W\_F{C}_{c,j}$$

### Crop yield data

Gridded crop yield data are available at 0.5° grid spacing in the recent *Global Dataset of Historical Yield* (GDHY) for major crops from 1981 to 2016^90^. This hybrid dataset merges agricultural census statistics and satellite remote sensing data. Crop yields are already weighted according to the respective crop fraction in the cell and estimates are available, consistently with the above-mentioned crop calendar dataset used as input, separately for the cropping systems and also for the overall crop category by weighting the single cropping systems. Here, the cropping system yields were considered and a linear detrending was applied to remove possible effects of improvements in the agricultural practices and technologies^14,16–18,20–23,26^. Then, a Standardized Yield Index (SYI) was derived, using the same approach previously described for SPEI in case of 12-month duration (as yields refer to yearly values), and gridded SYI were averaged within countries (Eq. 5).

5 $${SYI}_{c,cnt}=\frac{1}{n}{\sum }_{j=1}^{n}{SYI}_{j}$$

where *c* here is extended to represent the cropping system, not just the crop, and *n* is the number of coarser resolution (0.5°) pixels *j* with the cropping system *c*﻿ in the country *cnt*.

The use of SYI provided standardized yield values comparable among countries, i.e., regardless of the typical yield regime, and enabled classification into normal to anomalous lower or higher yield as in Table 3.

**Table 3:** SYI classification.

Value classification	Class description	Macro class
SYI ≤ − 2	Extremely lower yield (ELY)	Lower yield (LY)
− 2.0 < SYI ≤ − 1.5	Severely lower yield (SLY)	
− 1.5 < SYI ≤ − 1.0	Moderately lower yield (MLY)	
− 1.0 < SYI < 1.0	Normal yield (NY)	Normal yield (NY)
1.0 ≤ SYI < 1.5	Moderately higher yield (MHY)	Higher yield (HY)
1.5 ≤ SYI < 2.0	Severely higher yield (SHY)	
SYI ≥ 2.0	Extremely higher yield (EHY)	

### Data sampling and analysis

**Figure 5:**
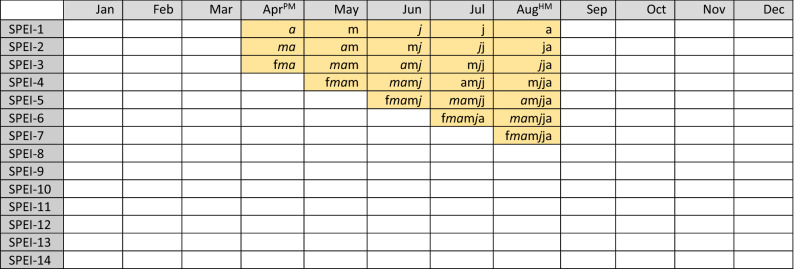
Example (yellow cells) of the sub-set of SPEI durations and timings considered if assuming harvest in August and a 5-month season length (i.e., planting in April). The superscripts PM and HM represent the planting and harvesting month, respectively. In each yellow cell, the small letters represent the consecutive months considered in the SPEI-*d* calculation and in particular: f = February, *m* = March, *a* = April, m = May, *j* = June, j = July, a = August.

Both SYI and SPEI have been calculated along 36 years and for the world’s countries for which described agricultural datasets were simultaneously available, i.e., countries without cultivated surface or yield/calendar data for one crop or cropping system have been excluded from the respective study (Table S3 in Supplementary material reports the harvested area calculated for each cropping system in each country). According to the cropping system’s calendar in each country, a subset of SPEI durations and timings was kept to cover the period from the harvesting month (HM) and back to cover up to two months before planting month (PM). An illustrative example is provided in Fig. 5.

**Figure 6:**
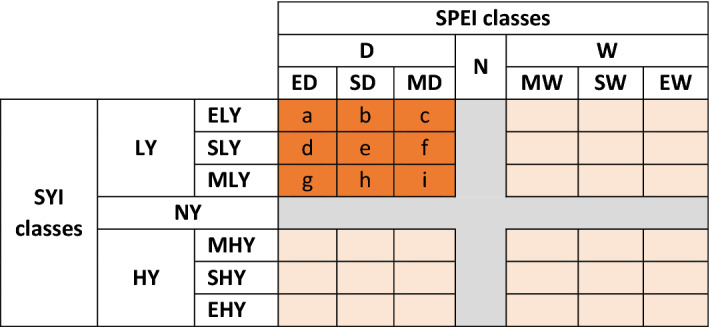
Contingency table among SPEI (columns) and SYI (rows) (macro)classes, see Tables 2 and 3 for acronyms in bold. Small letters in the orange side supported more in-depth analyses of the combination between LY and D macro classes.

To consider the role of *frequency* of drought, for each SPEI duration and timing, the occurrences for all the combinations between SPEI and SYI classes have been counted and organized in a contingency table like in Fig. 6.

### Climate and yield anomalous conditions

To consider the role of drought *frequency*, an index called *Asymmetry* (Eqs. 6–9) was calculated that, when greater than 50%, means that occurrences are skewed towards LY_D rather than towards other macro-classes. After some tests (not shown) with different minimums of sample size up to 177 (number of countries), showing no significant difference in results, a minimum of 36 values in each the contingency table was assumed enough to calculate the Asymmetry, foreseeing the possibility to have at least 1 count for each anomalous class combination in Fig. 6.

6 $${Asymmetry}_{LY\_DvsHY\_W}=100\%\times \frac{LY\_D}{LY\_D+HY\_W}$$

7 $${Asymmetry}_{LY\_DvsLY\_W}= 100\%\times \frac{LY\_D}{LY\_D+LY\_W}$$

8 $${Asymmetry}_{LY\_DvsHY\_D}= 100\%\times \frac{LY\_D}{LY\_D+HY\_D}$$

9 $$Asymmetry= mean\left(\frac{LY\_D}{LY\_D+HY\_W},\frac{LY\_D}{LY\_D+LY\_W},\frac{LY\_D}{LY\_D+HY\_D}\right)\quad \mathrm{only\,if,Eqs}. 6 \& 7 \& 8 > 50{\%}$$

Replacing *LY_D* with *HY_W* in Eqs. (6–9) allows replicating the analysis focusing on higher-than-normal yields when wetter periods occur, to have a more comprehensive view of affine yield and moisture anomalous conditions.

Then, still from each contingency table, the differences in the frequencies of macro-classes’ occurrences have been evaluated using Fisher's exact test (one-sided) in Real Statistics v7.4, distinguishing when Asymmetry for *LY_D* (Eq. 9) and for *HY_W* is greater than 50%. The null hypothesis in the test is that the differences in the frequencies are not significant.

To consider drought *magnitude*, for each duration and timing the differences between the SYI values in case of SPEI ≤ − 1 vs. SPEI > − 1 have been evaluated through the two-sided Wilcoxon rank sum test for independent samples using Real Statistics v7.4. In practice, we tested the null hypothesis that the statistical distributions of yield deviations from the normal are similar in case of drought (SPEI ≤ − 1) and non-drought (SPEI > − 1) conditions. The same was done for the differences between the SPEI values in case of SYI ≤ − 1 and SYI > − 1, i.e., testing the null hypothesis that the statistical distributions of moisture deviations is similar between cases of lower-than-normal (SYI ≤ − 1) and higher-than-normal (SYI > − 1) yields.

In all the above tests, the lower the *p*-values, the higher the evidence to reject the null hypotheses.

A SPEI duration vs. SPEI timing matrix was finally built by merging p-values from the three above mentioned tests (Fisher’s test and two Wilcoxon tests). The *p*-value aggregation was conducted using scaling of arithmetic, geometric and harmonic means of the three *p*-values following the approaches summarized in Vovk and Wang (^91^), and the most conservative results (worst combined *p*-value) was finally considered.

### Drought and yield loss conditions

Focus was then moved on the *LY_D* macro class to investigate its internal counts of co-occurrence between classes (*a*, *b*, *c*, *d*, *e*, *f*, *g*, *h*, *i* in Fig. 6). Two Dominance indices were considered to measure, for each duration and timing, how much frequently a magnitude class within the drought (D) macro class is associated with: equivalent or severer (higher) magnitude classes within the lower-than-normal yield (LY) macro class with respect to total classes’ co-occurrences under *LY_D* (Eq. 10); and only severer (higher) magnitude classes within the lower-than-normal yield (LY) macro class with respect to total co-occurrences of not analogous classes under *LY_D* (Eq. 11).

10 $${Dominance}_{LY\ge D}=100\% \times \frac{a+b+c+e+f+i}{a+b+c+d+e+f+g+h+i}$$

11 $${Dominance}_{LY>D}=100\% \times \frac{b+c+f}{b+c+d+f+g+h}$$

Symbols and “≥” and “>” refer to equal or higher and higher, respectively, severity magnitude of LY vs. D classes. To derive mean Dominance indices per cropping system, outcomes of Eqs. (10 and 11) were averaged across all the duration-timing contingency tables analysed; dominance was assumed to occur for values above 66.7% (from Eq. 10) and above 50% (from Eq. 11).

Finally, for each duration and timing, the differences in the whole sub-series of SYI ≤ − 1 when − 1.5 < SPEI ≤ − 1 (*c* + *f* + *i*), − 2 < SPEI ≤ − 1.5 (*b* + *e* + *h*) and SPEI ≤ − 2 (*a* + *d* + *g*) have been evaluated through a two-sided Wilcoxon rank sum test for independent samples. The same has been done comparing the differences in the whole sub-series of SYI ≤ − 1.5 when − 1.5 < SPEI ≤ − 1 (*c* + *f*), − 2 < SPEI ≤ − 1.5 (*b* + *e*) and SPEI ≤ − 2 (*a* + *d*) and in the whole sub-series of SYI ≤ − 2 when − 1.5 ≤ SPEI < − 1 (*c*), − 2 < SPEI ≤ − 1.5 (*b*) and SPEI ≤ − 2 (*a*).

## Supplementary Information

Supplementary Information 1.

Supplementary Information 2.

Supplementary Information 3.

## Data Availability

All dataset used in the manuscript are publicly available as reported in “Methods”. All new data generated from these sources, and related scripts or worksheets, are available from the corresponding author upon request.
