# Supplementary material for: Complex drought patterns robustly explain global yield loss for major crops
Source: Sci Rep. 2022 Apr 6;12:5792. doi: 10.1038/s41598-022-09611-0 (PMC8986840; doi:10.1038/s41598-022-09611-0)
Supplement: Supplementary file 1 — Supplementary Information 1. [file 41598_2022_9611_MOESM1_ESM.docx]

**Complex drought patterns robustly explain global yield loss for major crops**

**Monia Santini^1,*^, Sergio Noce^1^, Marta Antonelli^1,2^ and Luca Caporaso^1,3^**

^1^ Impacts on Agriculture, Forests and Ecosystem Services (IAFES) Division, Foundation Euro-Mediterranean Center on Climate Change (CMCC), 01100 Viterbo, Italy

^2^ Barilla Center for Food & Nutrition (BCFN) Foundation, 43121 Parma, Italy

^3^ European Commission – Joint Research Centre (JRC), 21027 Ispra, Italy

*corresponding author: Monia Santini, [monia.santini@cmcc.it](mailto:monia.santini@cmcc.it)

**Supplementary Information**

[Table S1. Average asymmetry. 2](#_Toc98060710)

[Table S2. List of countries. 3](#_Toc98060711)

[Figure S1. Frequency significance of co-occurring lower-than-normal both moisture and yield. 5](#_Toc98060712)

[Figure S2. Significance of differences in SYI magnitude under different SPEI intervals. 6](#_Toc98060713)

[Figure S3. Significance of differences in SPEI magnitude under different SYI intervals. 7](#_Toc98060714)

[Figure S4. Frequency significance of co-occurring higher-than-normal both moisture and yield. 8](#_Toc98060715)

[Figure S5. Spatial distribution of crop harvested areas. 9](#_Toc98060716)

[Table S3. Harvested areas per crop system and country. 10](#_Toc98060717)

[Table S4. Crop susceptibility per country and year. 17](#_Toc98060718)

[Table S5. Aggregated crop susceptibility per country and year. 24](#_Toc98060719)

[References 26](#_Toc98060720)

# Table S1. Average asymmetry.

Aggregated results per cropping system (first column) in terms of Asymmetry (fourth column, quantifying the average prevalence of the *LY_D* macro-class with respect to any other macro-class, see Eqs. 6-9 in Methods). Enter brackets, the share of duration-timing contingency tables presenting full Asymmetry is shown as calculated over the total number of combinations analysed (third column). The global statistics of growing season length is also reported in the second column.

| **Cropping system** | **Growing season length (in months)**  **mean±st.dev. [maximum]** | **N. of**  **contingency tables analysed** | **Asymmetry**  **[and % of contingency tables with full Asymmetry > 50%]** |  |
| --- | --- | --- | --- | --- |
|  |  |  |  |  |
| Maize main season | 5.1±1.2 [7.1] | 42 | 71% [95%] |  |
| Maize 2^nd^ season | 4.7±0.9 [5.8] | 25 | 66% [76%] |  |
| Rice main season | 5±0.9 [7.2] | 33 | 57% [48%] |  |
| Rice 2^nd^ season | 4.2±0.5 [5.4] | 12 | - |  |
| Soybean | 5±0.7 [6.1] | 33 | 67% [91%] |  |
| Wheat (spring) | 4.6±0.9 [7.2] | 25 | 67% [84%] |  |
| Wheat (winter) | 7.5±2.2 [11.6] | 85 | 63% [68%] |  |
| *Averag*e | | | *64% [76%]* |  |

# Table S2. List of countries.

List (with ID code) of the 177 countries considered in the analysis as extracted from the Database of Global Administrative Areas (GADM).

| **ID** | **Country name** | **ID** | **Country name** |
| --- | --- | --- | --- |
| 1 | Afghanistan | 69 | Egypt |
| 3 | Albania | 70 | El Salvador |
| 4 | Algeria | 71 | Equatorial Guinea |
| 7 | Angola | 72 | Eritrea |
| 11 | Argentina | 73 | Estonia |
| 12 | Armenia | 74 | Ethiopia |
| 14 | Australia | 75 | Falkland Islands |
| 15 | Austria | 77 | Fiji |
| 16 | Azerbaijan | 78 | Finland |
| 17 | Bahamas | 79 | France |
| 19 | Bangladesh | 80 | French Guiana |
| 21 | Belarus | 82 | French Southern Territories |
| 22 | Belgium | 83 | Gabon |
| 23 | Belize | 84 | Gambia |
| 24 | Benin | 85 | Georgia |
| 26 | Bhutan | 86 | Germany |
| 27 | Bolivia | 87 | Ghana |
| 29 | Bosnia and Herzegovina | 89 | Greece |
| 30 | Botswana | 90 | Greenland |
| 32 | Brazil | 94 | Guatemala |
| 35 | Brunei | 96 | Guinea |
| 36 | Bulgaria | 97 | Guinea-Bissau |
| 37 | Burkina Faso | 98 | Guyana |
| 38 | Burundi | 99 | Haiti |
| 39 | Cambodia | 101 | Honduras |
| 40 | Cameroon | 103 | Hungary |
| 41 | Canada | 104 | Iceland |
| 45 | Central African Republic | 105 | India |
| 46 | Chad | 106 | Indonesia |
| 47 | Chile | 107 | Iran |
| 48 | China | 108 | Iraq |
| 52 | Colombia | 109 | Ireland |
| 55 | Costa Rica | 111 | Israel |
| 56 | Cote d'Ivoire | 112 | Italy |
| 57 | Croatia | 113 | Jamaica |
| 58 | Cuba | 114 | Japan |
| 60 | Cyprus | 116 | Jordan |
| 61 | Czech Republic | 117 | Kazakhstan |
| 62 | Democratic Republic of the Congo | 118 | Kenya |
| 63 | Denmark | 120 | Kosovo |
| 64 | Djibouti | 121 | Kuwait |
| 66 | Dominican Republic | 122 | Kyrgyzstan |
| 67 | East Timor | 123 | Laos |
| 68 | Ecuador | 124 | Latvia |

| **ID** | **Country name** | **ID** | **Country name** |
| --- | --- | --- | --- |
| 125 | Lebanon | 198 | Saudi Arabia |
| 126 | Lesotho | 199 | Senegal |
| 127 | Liberia | 200 | Serbia |
| 128 | Libya | 202 | Sierra Leone |
| 130 | Lithuania | 205 | Slovakia |
| 131 | Luxembourg | 206 | Slovenia |
| 133 | Macedonia | 207 | Solomon Islands |
| 134 | Madagascar | 208 | Somalia |
| 135 | Malawi | 209 | South Africa |
| 136 | Malaysia | 210 | South Georgia and the South Sandwich Islands |
| 138 | Mali | 211 | South Korea |
| 142 | Mauritania | 212 | South Sudan |
| 145 | Mexico | 213 | Spain |
| 147 | Moldova | 215 | Sri Lanka |
| 149 | Mongolia | 216 | Sudan |
| 150 | Montenegro | 217 | Suriname |
| 152 | Morocco | 218 | Svalbard and Jan Mayen |
| 153 | Mozambique | 219 | Swaziland |
| 154 | Myanmar | 220 | Sweden |
| 155 | Namibia | 221 | Switzerland |
| 157 | Nepal | 222 | Syria |
| 158 | Netherlands | 223 | Taiwan |
| 159 | New Caledonia | 224 | Tajikistan |
| 160 | New Zealand | 225 | Tanzania |
| 161 | Nicaragua | 226 | Thailand |
| 162 | Niger | 227 | Togo |
| 163 | Nigeria | 230 | Trinidad and Tobago |
| 166 | North Korea | 231 | Tunisia |
| 168 | Norway | 232 | Turkey |
| 169 | Oman | 233 | Turkmenistan |
| 170 | Pakistan | 236 | Uganda |
| 172 | Palestina | 237 | Ukraine |
| 173 | Panama | 238 | United Arab Emirates |
| 174 | Papua New Guinea | 239 | United Kingdom |
| 175 | Paraguay | 240 | United States |
| 176 | Peru | 242 | Uruguay |
| 177 | Philippines | 243 | Uzbekistan |
| 179 | Poland | 244 | Vanuatu |
| 180 | Portugal | 246 | Venezuela |
| 181 | Puerto Rico | 247 | Vietnam |
| 182 | Qatar | 250 | Western Sahara |
| 183 | Republic of Congo | 251 | Yemen |
| 185 | Romania | 252 | Zambia |
| 186 | Russia | 253 | Zimbabwe |
| 187 | Rwanda |  |  |

| **a) maize main season**  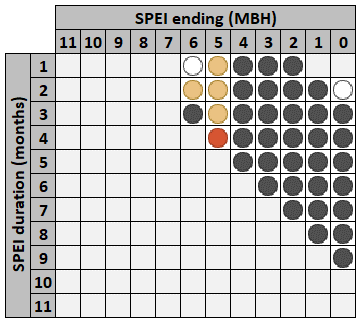 | **b) maize second season**  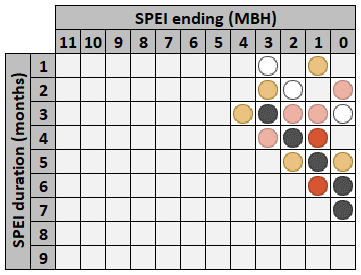 |
| --- | --- |
| **c) rice main season**  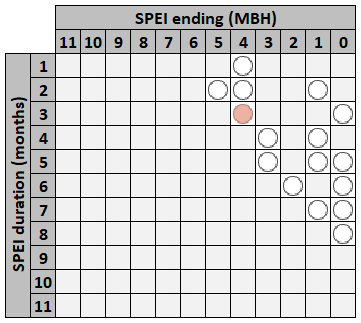 | **d) rice second season**  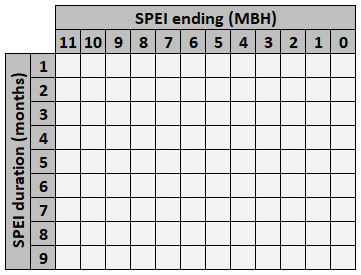 |
| **e) soybean**  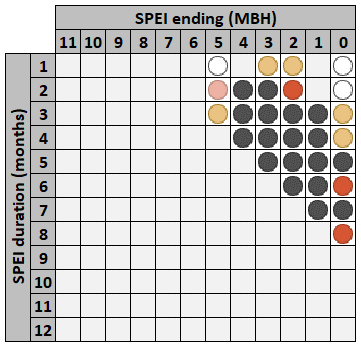 | **f) spring wheat**  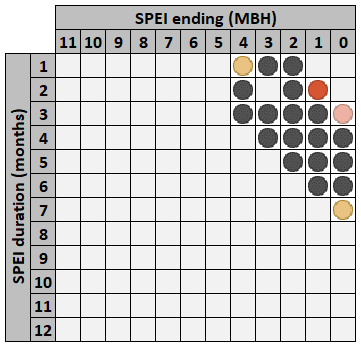 |
| **g) winter wheat**  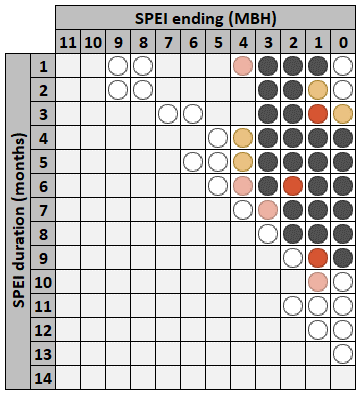 | **Legend**  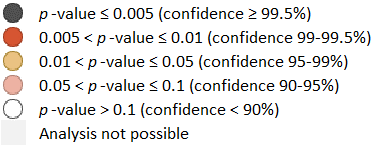 |

# Figure S1. Frequency significance of co-occurring lower-than-normal both moisture and yield.

SPEI duration-timing matrix of *p*-values from the Fisher exact test between anomalous macro-classes of contingency tables, showing the significance of the Asymmetry fully skewed towards the co-occurrence of lower-than-normal both yield and moisture. Grey cells represent excluded contingency tables, i.e., when the minimum sample to do the analysis is no reached or when there is no dominance of the *LY_D* macro-class over all the other macro-classes. Coloured symbols refer to *p*-value classification as reported in the legend. SPEI ending (in Months Before the Harvesting month, MBH) represents the timing.

| **a) maize main season**  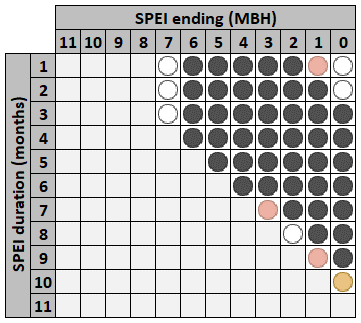 | **b) maize second season**  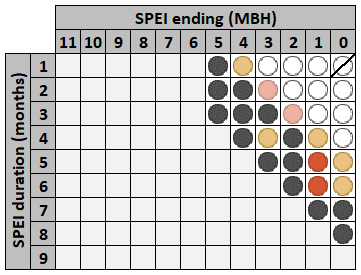 |
| --- | --- |
| **c) rice main season**  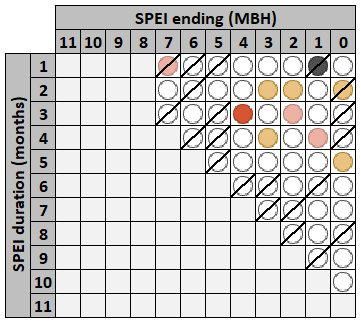 | **d) rice second season**  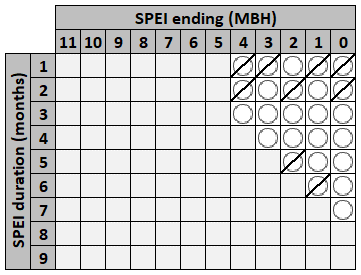 |
| **e) soybean**  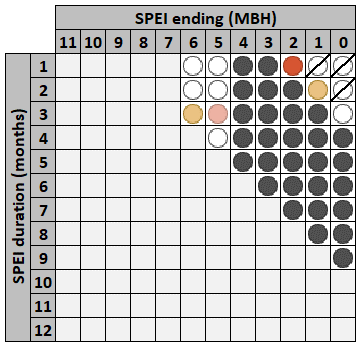 | **f) spring wheat**  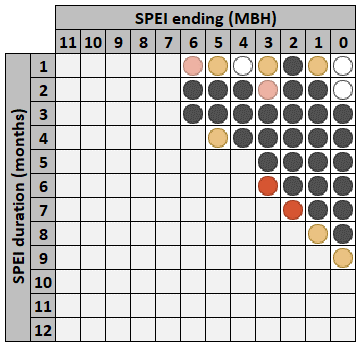 |
| **g) winter wheat**  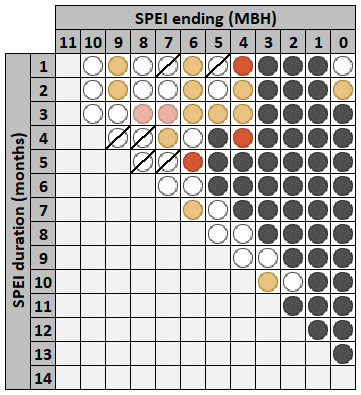 | **Legend**  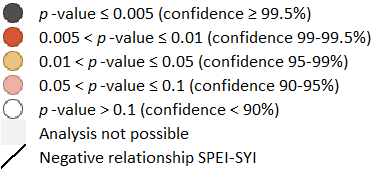 |

# Figure S2. Significance of differences in SYI magnitude under different SPEI intervals.

SPEI duration-timing matrix of *p*-values of the Wilcoxon rank sum test for independent sample made between the series of SYI values under SPEI ≤ -1 (drought conditions) vs. SPEI > -1. Coloured symbols refer to *p*-value classification as reported in the legend. Grey cells represent no minimum sample reached to do the analysis. Diagonal lines indicate the case of overall SYI higher under drought (drier-than-normal) conditions. SPEI ending (in Months Before the Harvesting month, MBH) represents the timing.

| **a) maize main season**  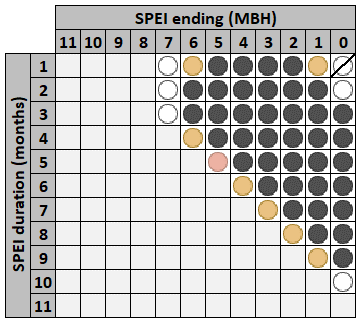 | **b) maize second season**  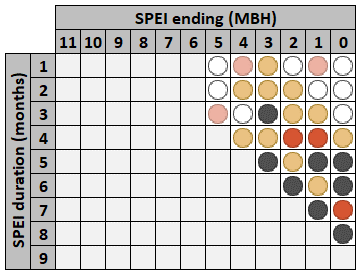 |
| --- | --- |
| **c) rice main season**  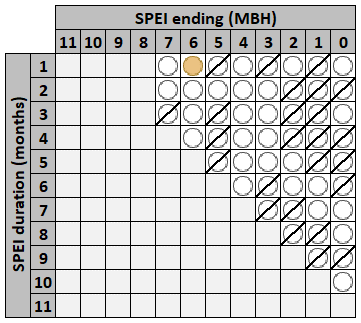 | **d) rice second season**  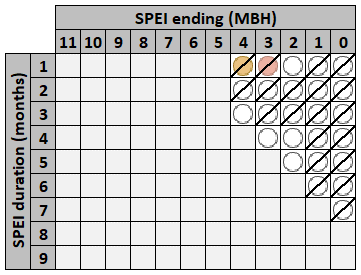 |
| **e) soybean**  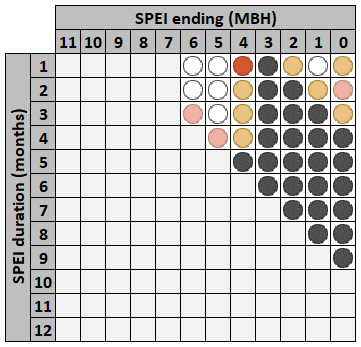 | **f) spring wheat**  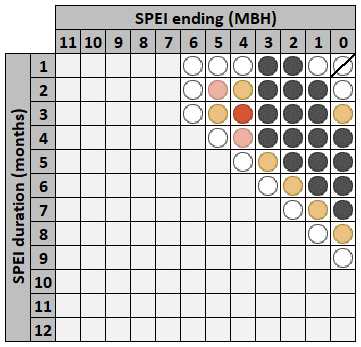 |
| **g) winter wheat**  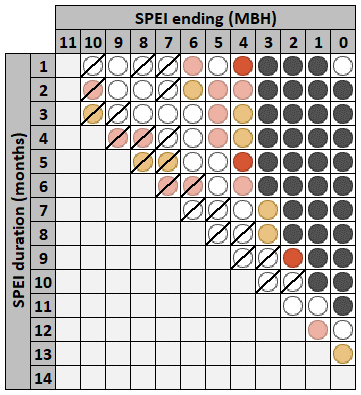 | **Legend**  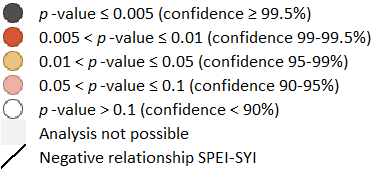 |

# Figure S3. Significance of differences in SPEI magnitude under different SYI intervals.

SPEI duration-timing matrix of *p*-values of the Wilcoxon rank sum test for independent sample made between the series of SPEI values under SYI ≤ -1 (yield lower-than-normal) vs. SYI > -1. Coloured symbols refer to *p*-value classification as reported in the legend. Grey cells represent no minimum sample reached to do the analysis. Diagonal lines indicate the case of SPEI on average found higher under the occurrence of lower-than-normal yields. SPEI ending (in Months Before the Harvesting month, MBH) represents the timing.

| **a) maize main season**  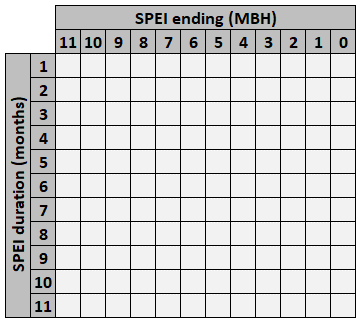 | **b) maize second season**  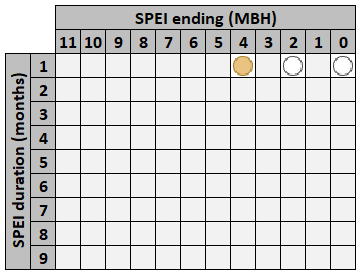 |
| --- | --- |
| **c) rice main season**  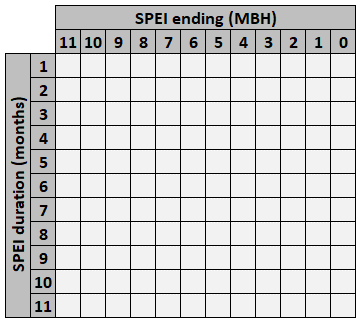 | **d) rice second season**  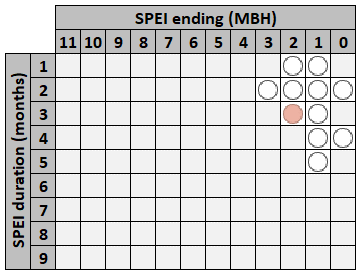 |
| **e) soybean**  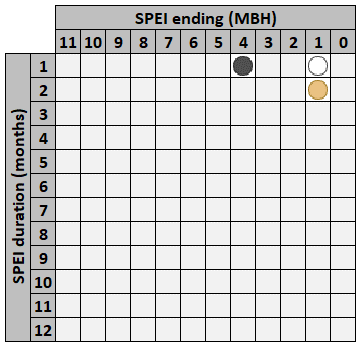 | **f) spring wheat**  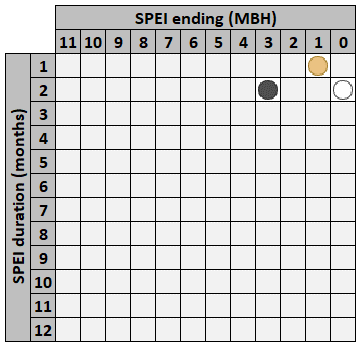 |
| **g) winter wheat**  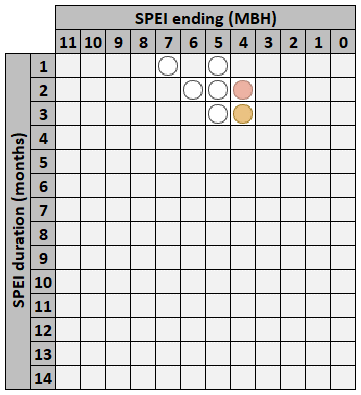 | **Legend**  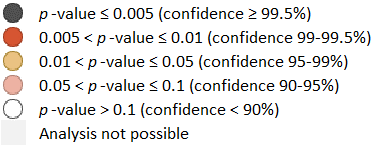 |

# Figure S4. Frequency significance of co-occurring higher-than-normal both moisture and yield.

SPEI duration-timing matrix of *p*-values from the Fisher exact test among anomalous macro-classes of contingency tables, showing the significance of the asymmetry fully skewed towards the co-occurrence of higher-than-normal both yield and moisture. Grey cells represent excluded contingency tables, i.e., when minimum sample to do the analysis is no reached or there is no dominance of the *HY_W* macro-class over all the other macro-classes. Coloured symbols refer to *p*-value classification as reported in the legend. SPEI ending (in Months Before the Harvesting month, MBH) represents the timing.

| 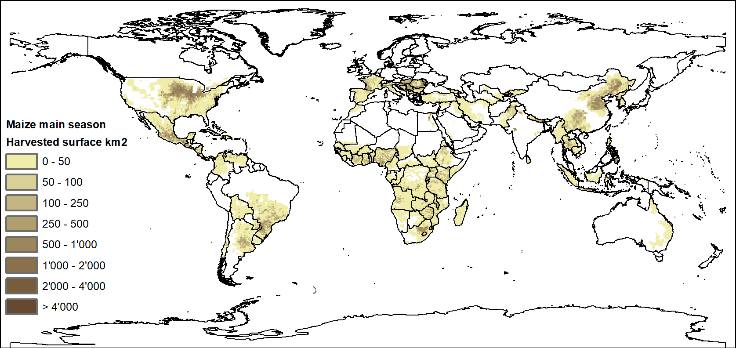 | 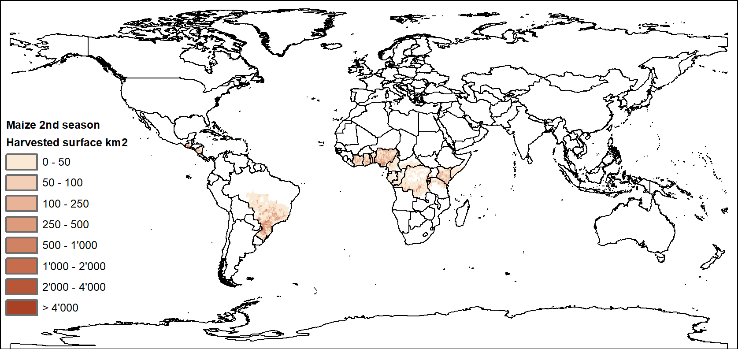 |
| --- | --- |
| 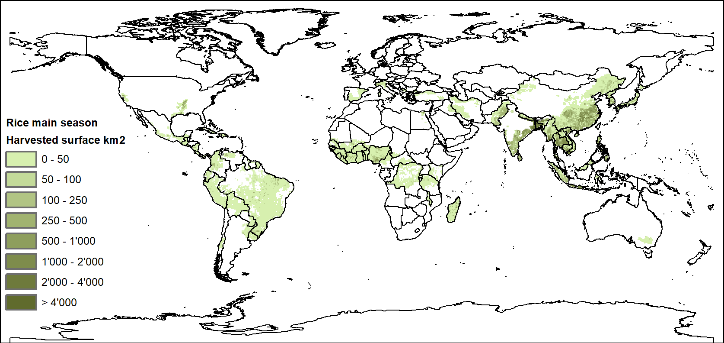 | 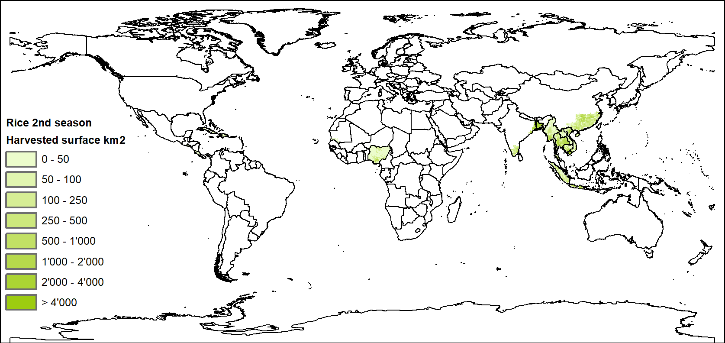 |
| 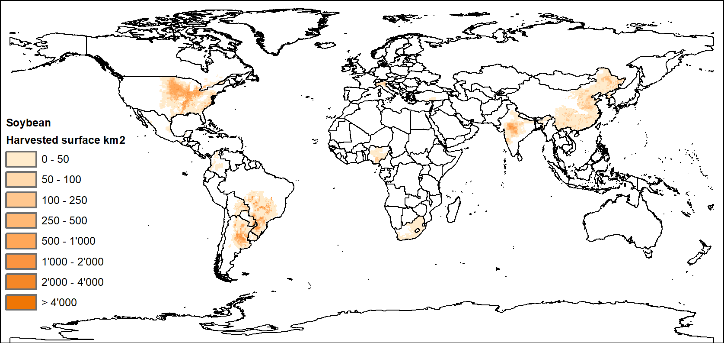 | 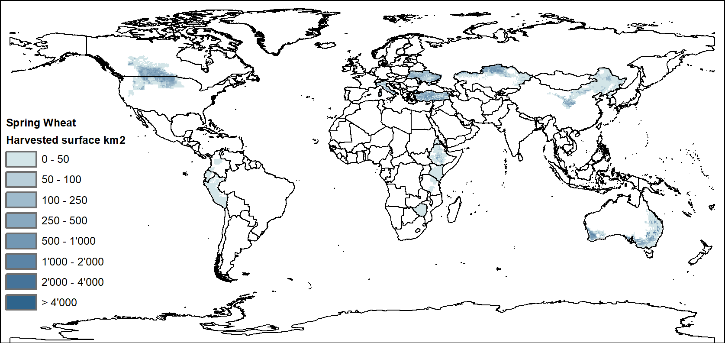 |
| 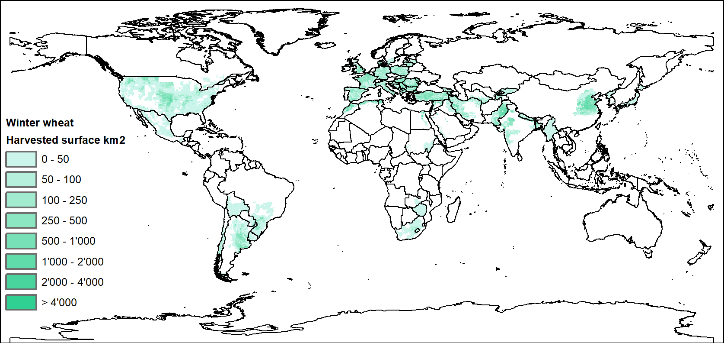 |  |

# Figure S5. Spatial distribution of crop harvested areas.

Maps showing the spatial distribution of surface (in km^2^) devoted to each crop system, derived from the cultivated cells for each crop system according to the crop yield database *(1)* multiplied by the fraction of harvested area of the related crop *(2)* and by the cell area calculated from *(3)*. A few inconsistencies with the maps in Figure 2 and with Table S3 can be due to spatial discrepancy among fraction cover, yield, and calendar datasets, and to simplified countries boundaries because of gridded spatial discretization.

# Table S3. Harvested areas per crop system and country.

Surface (in km^2^) devoted to each crop system per country, derived from the cultivated cells for each crop system according to the crop yield database *(1)* multiplied by the fraction of harvested area of the related crop *(2)* and by the cell area calculated from *(3)*. A few inconsistencies with the maps in Figure 2 and Figure S5 can be due to spatial discrepancy among fraction cover, yield, and calendar datasets, and to simplified countries boundaries because of gridded spatial discretization.

|  |  | **Harvested surface in km^2^** | | | | | | |
| --- | --- | --- | --- | --- | --- | --- | --- | --- |
| **ID** | **Country name** | **maize main** | **maize 2nd** | **rice main** | **rice 2nd** | **soybean** | **spring wheat** | **winter wheat** |
| 1 | Afghanistan | 52.456 |  |  |  |  |  | 1'398.815 |
| 3 | Albania | 444.515 |  |  |  |  |  | 702.586 |
| 4 | Algeria |  |  |  |  |  |  | 14'975.297 |
| 7 | Angola | 6'716.228 | 136.479 | 4.028 |  |  |  |  |
| 11 | Argentina | 27'772.594 | 744.810 | 2'185.616 |  | 90'431.129 |  | 55'863.892 |
| 12 | Armenia |  |  |  |  |  | 475.523 | 1'049.396 |
| 14 | Australia | 475.190 |  | 1'032.702 |  |  | 101'261.354 |  |
| 15 | Austria | 894.696 |  |  |  |  |  | 1'100.509 |
| 16 | Azerbaijan | 55.968 |  | 6.219 |  |  |  | 1'690.847 |
| 17 | Bahamas |  |  |  |  |  |  |  |
| 19 | Bangladesh |  |  | 86'781.457 | 86'781.457 |  |  | 5'184.390 |
| 21 | Belarus |  |  |  |  |  | 245.290 | 278.464 |
| 22 | Belgium | 40.446 |  |  |  |  |  | 499.452 |
| 23 | Belize | 23.627 |  |  |  |  |  |  |
| 24 | Benin | 3'019.612 | 3'019.612 | 190.285 | 73.508 | 0.871 |  |  |
| 26 | Bhutan |  |  | 10.295 | 10.295 | 0.345 |  |  |
| 27 | Bolivia | 2'674.936 | 10.354 | 1'371.683 |  | 5'742.437 | 0.833 | 1'397.423 |
| 29 | Bosnia and Herzegovina | 1'794.883 |  |  |  |  |  | 837.823 |
| 30 | Botswana | 364.372 |  |  |  | 0.070 | 1.884 | 2.020 |
| 32 | Brazil | 90'030.929 | 89'887.426 | 26'898.568 |  | 126'544.194 |  | 16'855.641 |
| 35 | Brunei |  |  | 2.633 |  |  |  |  |
| 36 | Bulgaria | 3'849.018 |  | 2.253 |  |  | 361.988 | 10'903.669 |
| 37 | Burkina Faso | 2'038.042 | 54.109 | 296.028 |  |  |  |  |
| 38 | Burundi | 1'293.216 | 1'293.216 | 96.311 |  |  |  |  |
| 39 | Cambodia | 544.198 |  | 19'199.039 | 19'199.039 |  |  |  |
| 40 | Cameroon | 2'961.854 | 2'961.854 | 199.447 | 43.486 | 13.399 |  |  |
| 41 | Canada | 5'600.997 |  |  |  | 5'126.403 | 98'718.294 | 1'569.090 |
| 45 | Central African Republic | 550.395 | 533.463 | 96.792 |  |  |  |  |
| 46 | Chad | 906.816 | 244.992 | 185.778 |  |  |  |  |
| 47 | Chile | 761.212 |  | 206.899 |  |  | 0.228 | 2'379.497 |
| 48 | China | 208'313.929 |  | 284'976.239 | 152'399.629 | 72'535.197 | 36'927.005 | 163'275.821 |
| 52 | Colombia | 5'924.353 |  | 4'479.260 |  | 298.693 | 166.503 |  |
| 55 | Costa Rica | 35.618 | 35.618 | 164.504 | 164.504 |  |  |  |
| 56 | Cote d'Ivoire | 7'247.684 | 7'247.684 | 4'405.027 |  |  |  |  |
| 57 | Croatia | 4'460.410 |  |  |  |  |  | 2'462.351 |
| 58 | Cuba | 626.748 | 626.748 | 928.046 | 928.046 |  |  |  |
| 60 | Cyprus |  |  |  |  |  |  |  |
| 61 | Czech Republic |  |  |  |  |  |  | 596.852 |
| 62 | Democratic Republic of the Congo | 17'181.089 | 16'602.788 | 4'754.455 |  |  |  |  |
| 63 | Denmark |  |  |  |  |  |  | 2'127.439 |
| 64 | Djibouti |  |  |  |  |  |  |  |
| 66 | Dominican Republic | 102.407 | 102.407 | 532.327 | 532.327 |  |  |  |
| 67 | East Timor |  |  |  |  |  |  |  |
| 68 | Ecuador | 119.653 |  | 2'416.376 |  |  | 239.039 |  |
| 69 | Egypt | 8'277.509 |  | 4'675.286 |  |  |  | 8'893.211 |
| 70 | El Salvador | 1'117.406 | 1'117.406 | 20.630 | 20.630 |  |  |  |
| 71 | Equatorial Guinea |  |  |  |  |  |  |  |
| 72 | Eritrea | 143.231 |  |  |  |  | 172.501 |  |
| 73 | Estonia |  |  |  |  |  |  |  |
| 74 | Ethiopia | 11'886.927 | 421.928 | 0.012 |  |  | 7'720.227 | 124.862 |
| 75 | Falkland Islands |  |  |  |  |  |  |  |
| 77 | Fiji |  |  |  |  |  |  |  |
| 78 | Finland |  |  |  |  |  |  |  |
| 79 | France | 16'108.707 |  | 3.577 |  | 0.668 | 28.985 | 44'115.224 |
| 80 | French Guiana |  |  | 17.660 |  |  |  |  |
| 82 | French Southern Territories |  |  |  |  |  |  |  |
| 83 | Gabon | 98.917 | 98.917 |  |  |  |  |  |
| 84 | Gambia | 55.929 |  | 14.331 |  |  |  |  |
| 85 | Georgia | 1'690.310 |  |  |  |  | 139.922 | 1'027.131 |
| 86 | Germany | 209.953 |  |  |  |  |  | 24'377.441 |
| 87 | Ghana | 6'341.453 | 6'341.453 | 1'193.195 |  |  |  |  |
| 89 | Greece | 500.407 |  | 1.879 |  |  | 331.289 | 1'272.188 |
| 90 | Greenland |  |  |  |  |  |  |  |
| 94 | Guatemala | 5'655.749 | 5'655.413 | 127.845 | 17.082 |  |  |  |
| 96 | Guinea | 808.589 | 190.687 | 4'546.847 |  |  |  |  |
| 97 | Guinea-Bissau | 89.926 |  | 220.083 |  |  |  |  |
| 98 | Guyana | 0.083 |  | 216.759 |  |  |  |  |
| 99 | Haiti | 423.233 | 423.233 | 353.401 | 353.401 |  |  |  |
| 101 | Honduras | 395.424 | 395.424 | 10.337 | 5.175 |  |  |  |
| 103 | Hungary | 10'267.812 |  |  |  |  | 127.161 | 10'689.433 |
| 104 | Iceland |  |  |  |  |  |  |  |
| 105 | India | 12'786.469 |  | 218'930.432 | 81'769.546 | 63'639.877 |  | 52'609.078 |
| 106 | Indonesia | 13'348.703 |  | 47'875.178 | 46'316.573 |  |  |  |
| 107 | Iran | 1'491.482 |  | 5'250.415 |  |  | 103.437 | 47'170.974 |
| 108 | Iraq | 732.490 |  | 543.078 |  |  | 17.755 | 1'211.354 |
| 109 | Ireland |  |  |  |  |  |  | 1.750 |
| 111 | Israel | 44.629 |  |  |  |  |  | 1'069.754 |
| 112 | Italy | 9'303.087 |  | 2'578.099 |  | 2'045.685 | 11'549.161 | 11'549.161 |
| 113 | Jamaica |  |  |  |  |  |  |  |
| 114 | Japan |  |  | 7'070.742 |  |  |  | 999.349 |
| 116 | Jordan | 11.437 |  |  |  |  |  | 189.335 |
| 117 | Kazakhstan | 134.382 |  | 4.820 |  |  | 95'738.626 |  |
| 118 | Kenya | 14'796.988 | 14'796.988 | 64.383 |  |  | 1'330.357 | 58.872 |
| 120 | Kosovo | 1'456.695 |  |  |  |  |  | 799.955 |
| 121 | Kuwait |  |  |  |  |  |  |  |
| 122 | Kyrgyzstan | 545.178 |  |  |  |  |  | 4'497.458 |
| 123 | Laos | 148.324 |  | 5'942.301 | 5'942.301 | 4.362 |  |  |
| 124 | Latvia |  |  |  |  |  |  | 1'171.059 |
| 125 | Lebanon |  |  |  |  |  |  | 5.867 |
| 126 | Lesotho | 1'213.356 |  |  |  | 12.710 |  | 248.838 |
| 127 | Liberia |  |  | 828.619 |  |  |  |  |
| 128 | Libya |  |  |  |  |  |  |  |
| 130 | Lithuania |  |  |  |  |  |  | 4'247.413 |
| 131 | Luxembourg |  |  |  |  |  |  | 24.437 |
| 133 | Macedonia | 371.352 |  | 49.011 |  |  |  | 1'391.671 |
| 134 | Madagascar | 1'020.848 |  | 8'944.028 |  |  |  |  |
| 135 | Malawi | 7'774.232 |  | 361.613 |  |  | 15.437 |  |
| 136 | Malaysia | 6.829 |  | 2'302.725 | 72.638 |  |  |  |
| 138 | Mali | 1'587.841 | 6.353 | 2'305.098 | 0.319 |  |  |  |
| 142 | Mauritania | 78.265 |  | 217.025 | 217.025 |  |  |  |
| 145 | Mexico | 69'025.998 | 1'637.355 | 732.733 |  | 353.803 |  | 3'404.322 |
| 147 | Moldova | 4'514.469 |  |  |  |  | 1'750.134 | 3'804.050 |
| 149 | Mongolia | 7.149 |  | 1.112 |  | 3.071 | 42.014 |  |
| 150 | Montenegro | 1'199.843 |  |  |  |  |  | 658.903 |
| 152 | Morocco | 1'198.657 |  |  |  |  |  | 21'871.377 |
| 153 | Mozambique | 8'812.044 |  | 14.329 |  |  | 0.801 | 0.364 |
| 154 | Myanmar | 1'928.133 |  | 44'164.625 | 44'164.625 | 57.912 |  | 726.466 |
| 155 | Namibia | 276.873 |  |  |  |  |  |  |
| 157 | Nepal | 6'044.050 |  | 10'482.649 |  | 39.031 |  | 4'456.309 |
| 158 | Netherlands |  |  |  |  |  |  | 125.135 |
| 159 | New Caledonia |  |  |  |  |  |  |  |
| 160 | New Zealand |  |  |  |  |  |  |  |
| 161 | Nicaragua | 3'411.372 | 3'411.372 | 772.013 | 26.085 |  |  |  |
| 162 | Niger | 9.419 | 7.882 | 161.611 | 2.765 |  |  |  |
| 163 | Nigeria | 35'623.652 | 35'623.652 | 22'943.897 | 22'943.897 | 4'015.856 |  |  |
| 166 | North Korea | 2'523.453 |  | 2'691.692 |  | 1'104.315 | 223.826 | 294.724 |
| 168 | Norway |  |  |  |  |  |  |  |
| 169 | Oman |  |  |  |  |  |  |  |
| 170 | Pakistan | 8'354.867 |  | 22'435.656 |  |  |  | 79'235.971 |
| 172 | Palestina |  |  |  |  |  |  | 144.135 |
| 173 | Panama | 232.052 | 88.822 | 274.152 | 70.359 |  |  |  |
| 174 | Papua New Guinea |  |  |  |  |  |  |  |
| 175 | Paraguay | 3'376.320 | 223.585 | 291.006 |  | 9'269.331 |  | 1'871.869 |
| 176 | Peru | 14.096 |  | 2'770.839 |  |  | 1'269.587 | 31.205 |
| 177 | Philippines | 10'527.237 |  | 15'519.698 |  |  |  |  |
| 179 | Poland |  |  |  |  |  | 469.808 | 21'853.530 |
| 180 | Portugal | 232.757 |  | 41.639 |  |  |  | 411.708 |
| 181 | Puerto Rico |  |  |  |  |  |  |  |
| 182 | Qatar |  |  |  |  |  |  |  |
| 183 | Republic of Congo | 64.612 | 64.612 | 0.377 |  |  |  |  |
| 185 | Romania | 31'133.693 |  |  |  |  | 762.182 | 20'903.677 |
| 186 | Russia | 28.027 |  | 25.171 |  | 13.689 | 16'007.105 | 365.058 |
| 187 | Rwanda | 952.268 | 952.268 | 4.823 |  |  |  |  |
| 198 | Saudi Arabia | 28.946 |  |  |  |  |  | 1'846.836 |
| 199 | Senegal | 792.938 |  | 444.777 | 48.435 |  |  |  |
| 200 | Serbia | 9'808.685 |  |  |  |  |  | 5'386.513 |
| 202 | Sierra Leone | 45.626 |  | 1'215.078 |  |  |  |  |
| 205 | Slovakia | 409.521 |  |  |  |  | 4.462 | 1'453.224 |
| 206 | Slovenia | 305.309 |  |  |  | 0.004 | 79.108 | 247.345 |
| 207 | Solomon Islands |  |  |  |  |  |  |  |
| 208 | Somalia | 1'480.287 | 1'480.287 |  |  |  | 0.890 |  |
| 209 | South Africa | 29'736.827 |  |  |  | 1'008.833 | 0.636 | 5'639.625 |
| 210 | South Georgia and the South Sandwich Islands |  |  |  |  |  |  |  |
| 211 | South Korea | 90.864 |  | 5'518.057 |  |  |  | 4.316 |
| 212 | South Sudan | 225.364 | 16.113 | 0.991 |  |  | 38.216 | 102.755 |
| 213 | Spain | 4'229.422 |  | 886.458 |  |  |  | 20'890.798 |
| 215 | Sri Lanka | 156.339 |  | 3'593.330 |  |  |  |  |
| 216 | Sudan | 50.992 |  |  |  |  | 66.881 | 835.227 |
| 217 | Suriname |  |  | 230.762 |  |  |  |  |
| 218 | Svalbard and Jan Mayen |  |  |  |  |  |  |  |
| 219 | Swaziland | 1'076.243 |  |  |  |  |  | 0.643 |
| 220 | Sweden |  |  |  |  |  |  | 842.858 |
| 221 | Switzerland | 82.549 |  |  |  |  |  | 74.400 |
| 222 | Syria | 445.423 |  |  |  | 3.419 | 2'615.299 | 12'557.131 |
| 223 | Taiwan |  |  |  |  |  |  |  |
| 224 | Tajikistan | 49.476 |  |  |  |  |  | 3'066.336 |
| 225 | Tanzania | 12'619.769 | 5'368.553 | 1'767.067 |  |  | 570.357 |  |
| 226 | Thailand | 11'794.847 |  | 95'247.483 | 95'247.483 |  |  | 0.145 |
| 227 | Togo | 2'649.918 | 2'649.918 | 330.953 |  |  |  |  |
| 230 | Trinidad and Tobago |  |  |  |  |  |  |  |
| 231 | Tunisia |  |  |  |  |  |  | 5'685.965 |
| 232 | Turkey | 4'500.330 |  | 215.869 |  | 128.574 | 82'037.998 | 82'037.998 |
| 233 | Turkmenistan | 118.495 |  | 26.706 |  |  |  | 5'783.398 |
| 236 | Uganda | 9'640.196 | 4'614.625 | 214.860 |  |  | 38.286 | 2.151 |
| 237 | Ukraine | 882.140 |  |  |  |  | 49'172.752 | 3'280.029 |
| 238 | United Arab Emirates |  |  |  |  |  |  |  |
| 239 | United Kingdom |  |  |  |  |  |  | 12'971.383 |
| 240 | United States | 285'465.242 |  | 12'667.564 |  | 287'737.752 | 89'278.981 | 175'884.604 |
| 242 | Uruguay | 333.872 | 37.937 | 1'189.784 |  | 95.674 |  | 1'164.717 |
| 243 | Uzbekistan | 404.623 |  |  |  |  | 334.091 | 3'887.436 |
| 244 | Vanuatu |  |  |  |  |  |  |  |
| 246 | Venezuela | 3'944.715 |  | 1'526.952 |  |  |  |  |
| 247 | Vietnam | 689.356 |  | 47'627.651 | 47'627.651 | 228.847 |  |  |
| 250 | Western Sahara |  |  |  |  |  |  |  |
| 251 | Yemen |  |  |  |  |  |  |  |
| 252 | Zambia | 5'386.711 |  | 6.935 |  |  | 0.174 | 117.604 |
| 253 | Zimbabwe | 11'058.302 |  |  |  | 18.231 | 390.831 | 390.831 |

# Table S4. Crop susceptibility per country and year.

Counts, for each country and year, of all duration-timing combinations with SPEI ≤ -1 in case of SYI ≤ -1 for: a) maize main season; b) maize second season; c) rice main season; d) rice second season; e) soybean; f) spring wheat; g) winter wheat. Empty cells represent country-year combination for which SYI > -1. Light to dark tones represent increasing counts according to colors used in Figure 3 for each cropping system. Countries (rows) and years (columns) are flagged in case they present a significantly different counts’ distribution with respect to that of all countries and all years, respectively, under the Wilcoxon rank sum test for independent sample: * if 0.05 < *p*-value ≤ 0.1 (confidence 90-95%); ** 0.01 < *p*-value ≤ 0.05 (confidence 95-99%); *** *p*-value ≤ 0.01 (confidence ≥ 99%).

# Table S5. Aggregated crop susceptibility per country and year.

Cumulated counts across all cropping systems, for each country and year, of all duration-timing combinations with SPEI ≤ -1 in case of SYI ≤ -1. Empty cells represent country-year combination for which SYI > -1. Light to dark tones represent increasing counts. Countries and years are flagged in case they present a significantly different counts’ distribution with respect to that of all countries and all years, respectively, under the Wilcoxon rank sum test for independent sample: * if 0.05 < *p*-value ≤ 0.1 (confidence 90-95%); ** 0.01 < *p*-value ≤ 0.05 (confidence 95-99%); *** *p*-value ≤ 0.01 (confidence ≥ 99%).

# References

1. Iizumi, T. & Sakai, T. The global dataset of historical yields for major crops 1981–2016. *Scientific Data* **7**, 97 (2020).
2. Monfreda, C., Ramankutty, N. & Foley, J. A. Farming the planet: 2. Geographic distribution of crop areas, yields, physiological types, and net primary production in the year 2000. *Global Biogeochemical Cycles* **22**, GB1022 (2008).
3. Santini, M., Taramelli, A. & Sorichetta, A. ASPHAA: a GIS-based algorithm to calculate cell area on a latitude-longitude (geographic) regular grid. *Transactions in GIS* **14**(3), 351-377 (2010).
