# Supplementary material for: Complex drought patterns robustly explain global yield loss for major crops
Source: Sci Rep. 2022 Apr 6;12:5792. doi: 10.1038/s41598-022-09611-0 (PMC8986840; doi:10.1038/s41598-022-09611-0)
Supplement: Supplementary file 2 — Supplementary Information 2. [file 41598_2022_9611_MOESM2_ESM.pdf]

a)	Year	1981	1982	1983	1984	1985	1986	1987	1988	1989	1990	1991	1992	1993	1994	1995	1996	1997	1998	1999	2000	2001	2002	2003	2004	2005	2006	2007	2008	2009	2010	2011	2012	2013	2014	2015	2016
Country				**									***								***			*													
1																			0	0	0	15																	
3													2	3	0																							
7										1				0	0	0											0	0									
11	**									32							8													28							
14																											0										
15													22																								
16										15																											
22	**															23	29																		11	5
23						16			0	0						1																1		0	9	21	
24				18																											0			7		19	
27																		0							0												
29	**								3												34			33				12					17				
30					6	5							24	7					1	0																		
32												0														1	2										
36														0			11		1		24	0						13									
37				8	15			3																				2				4					
38																								3		2												
39														23				25	2				3								3						
40			0	9										0	0																							
41									17				2								1	19	8										4				
45			0	10	0																																
46																						6	13														
47			27	14																								0									
52																	0	11	0	0														4			
55											0	0					0												0						0	1	
56							0	11	1	2																						0			0	29	0
57	**																				34			33				8					14				
58												0	12	15	3	0														13	0						
66															0			15	0		15																
68																	14		0	1			8														
69																																					0	0
70																			0	0											0		4				
71		0				3	0			1						1													0					0	4		
72												0									0			0	0				2								
74															1									5	14													
79					3						17													19										0		3	10
83																							13															
85																			2		14	0									0						
86					2																			25			5				1					5	
87				13																																		
89																			4																			
94																		4							6	0	0										
96									2	0																			0									
97																															3	11		0	12	12	6
98				3																							0	0		1						
99									0		14				2							1			4								3				
101			6									15							0													0						
103	***										6		18	22	1						33			30				6					26				
106																		0																		
107																																0	0				
108																					4																	
111			6	6			9																												1	0	
112																								29									0				
117															0	8	0	0																		
118					31												5													3						
120	**								12		20		3	24														5					17				
122															5	10	0	0																		
123																						1														
126													28			23												2									
127								0	2	3																										20	
133														13							20	3														
134													0						1	0	2	1	0														
135													24													8											0
136																		3																		
138																						4	21									9				
142				12																			12				1										
145										3																										
147	*												8		8						15							24					4				
149				0							0								0										0							
150	***								3		20		3	14							30			30				17					22				
152														0		7				3		19				16										
153												5	26														6								0			
154																			0	0	0															
155													20				29		21	7	0			0	0												
161		0	1	11								0																								5	
162					17																															
163																																		5			
166																1	4	12	0	0	12																
170																					7	4	16	0														
173																							3														
175																					30					5		0		9							
176													29		0	4		0		0																		
177																	2				0		0														
180													8	2																							
183			0								0		23	0	0																						
185													2								20							12					4				
186																			0			0															
187																								5		5		2									
198																		0	1	1																	
199																			3				16					1				3					
200	***										16		7	23							36			29				8					20				
202							8																													0	0
205	**										7		22	24	1						22			29				6									
206	*												18																				17			
208			0												10				0								0		0							
209													30			3			4	5								21									
211												1		0					0	0	12		2	0													
212															0				0					0													
213														1																						
215																				6	0	4	17	0		0	1	1									
216																																		0			
219				35								46										10	1				0									
221					17											0								27							3			0		14	2
222																																			0	0	
224															0	2					12																	
225																								3		0				0							
227							0	16											7														9			
232																						4	0														
233																	0	0	0	0																	
236																	0	4							0	0	1	0									
237													9								1							10					5				
240				0					4																							0				
242	*														0						32								29		28					
243																				0	12	14															
246																																					28	0
247																													0									
251																			0	0								0									
252													29						4	0		0	7														
253	**			23	8			24					27			9							8			1			4							

b)	Year	1981	1982	1983	1984	1985	1986	1987	1988	1989	1990	1991	1992	1993	1994	1995	1996	1997	1998	1999	2000	2001	2002	2003	2004	2005	2006	2007	2008	2009	2010	2011	2012	2013	2014	2015	2016
Country					**								*																								
7															0						0					0				0							0
11					26																													4			14
24	**														0					0					0												
27															0						0					1	1										0
32				0																																	
37																								1		13											
38				0									19	0	1																				8		
40			2	0	18																								13	0							
45														0											0												
46				0								0								2		6															
55								0		4																										3	14
56														1	0															7	2					0	
58						0																															
62													1		5						2	1															
66																			0	0	0				2		0										
70					0													0					0	0	0	0											0
71																		4									13										
74	*																						2		9												
83				16	26								3																								
87	**																								4	2	0										
94								0	0	0				0															0	0							
96								0			14		3									1											3				
99			0									0							0	0																	
101					10													26																			
118	**						0	0	0	0																						0			0		
127													0	0	0							0	0		0	0						0					
138		0																						0	0	0	0										
145			8	0								0							0	0																	
161					0	0					0	0																									
162					26	0						0	5	0																							
163				0								0					0	0		0		0		0													
173																					2		0			12		2		23							4
175	*		1								0				4										17												1
183																								2		15											
187			5												2			16									0		0								
208																											0										
212															16			19						0		0											
225							0	0																										2			
227																		0							4		13	0									
236															0						0					0	0		0								0
242															0						0					0	0		0								0

c)	Year	1981	1982	1983	1984	1985	1986	1987	1988	1989	1990	1991	1992	1993	1994	1995	1996	1997	1998	1999	2000	2001	2002	2003	2004	2005	2006	2007	2008	2009	2010	2011	2012	2013	2014	2015	2016
Country				**										***																						**	
11												0																								0						
14																												8												
15				3			1																1									3						
16	*									24																				4								
19																																				1				
24														0																			0						
26		8						1	1														2		6						0					5	4
27																					1										0								
32																				0																		
35								9	0																				2								1		34
36										0	1	0	1						0	1																	
37								0																											6				
38																				0																		
39	**												2	8	25											7														
40		0	0		21																			0	0	1			0									
45																																	0						
46			0	1	10	3									7																								
47																				5	0												0						
52																																			1	0		4			
55			2																									0									7	10			
56														11		1	0		5																0					
58														9	14	4	0	0		18													8	0						
62															0	31																								
66																1				1						7	0				0								
68								6																														0	
70																			0		0	0	0		4									3						
74																					0							0	0	0	0	1							
79						1																															1			17
80			2	7																	0					1	0											
87			4				0			2									12											0									
89			0	1										0			0																					
94																			1	0						19													
96																																						0	3	3	1
97		0	3	23																	0		4	23		14												
98									4		0			9																			1						
99															2	2									1	13		4								4				
101																								2		5									2						
108	**																			15	14																		
112																							0																
114																												1					0										0
117				0			3													5				16		1	2	4										1			
118																																		2	4						
120																															2				1						
123																																										18	0
127												3	3											0	0	0													
133																																			1						
134																				3		9	3	0	0								0					
135														32													0	4												
136								2	0																												0		11
138						22																					0						3			9				
142															0																							
145																														1	1									
149				0							0								0																0				
153														28														0													
154																				13																		
157			2				0																															
161																							15	2	0			0											
162																																	0	0	3					
163																							0	0						0										
166	*																	4	12			12									5							1				
173																	1									0													
175																													7	10	0										
177																				20	0	0																
180	**												8					7													20								
183														9		6	0																							
186																				1						3														
187																				5	0										3									
199																				5					23					0	0										
202																							0	0	0	20	0	20										
211		3												0											0					0										
212																			0	0	0	0	0																
215						5											0																						
217																												2												
225			3										3						6	0									0			3	0						
226	*														23																									6	
227																																								0	22	1
232																	2		0																			
233								2																	18	0				2									
236																											0				0								
240																				0	14																		
242											0								0						0													
246								0	1	0	3																										25	6
247																																									0
252	*												17													8										

d)	Year	1981	1982	1983	1984	1985	1986	1987	1988	1989	1990	1991	1992	1993	1994	1995	1996	1997	1998	1999	2000	2001	2002	2003	2004	2005	2006	2007	2008	2009	2010	2011	2012	2013	2014	2015	2016
Country																																**					
19	*														1																		2			4	
24															0						0															5	
26			0	0		0							0		0						0																
39														1	4										9											0	
40					4																		0	0	0	0		0									
48		0							2									0																			
55	**		16																					0								7	17				
58	*												0	0												10				10		23					
66									2										2			1					0										
70																					0		0	0	1	0					4						
94						0		0									0	0	0					0	0	0		0									
99												12		4					0			1															
101																					0		0		0	0					0						
105															0																						
106																										0					0						
123																																				0	3
136					0	0																								0						0	0
138									0	0	0			0	0	0	0																				
142									0		0			0	0	0	0																				
154															0				0																	8	
161			0	0																0		0				0	0										
163															1						15		0		0												
173			0																	0		0		0		0							0				
199															0	0																					
226	**																																			6	14
247															0	0																					0

e)	Year	1981	1982	1983	1984	1985	1986	1987	1988	1989	1990	1991	1992	1993	1994	1995	1996	1997	1998	1999	2000	2001	2002	2003	2004	2005	2006	2007	2008	2009	2010	2011	2012	2013	2014	2015	2016
Country													**																								
11																		6												23							
15					3								20																		0						
19							5	0													2										1						
24															0		1								0				0	1				18			2
26	*						5	12																	4					23					15	9	
27						1							0							1							1	0	0								
30	*							15					33			4								6				11									
32												0													0	2	0										
40																														5							
41				0																		17		0													
48			0							0																		0									
52																									0	0	0				9						
79					2					18			0											18										0			8
105								13													11															4	
112				11																				29									0				
123										4	1				0													0	0								
126			12					12					26		1	21	0											2									
145																0	0														1						
149											0		0															8	0					0			
153			4					11					38		38		0																				
154			0																																		
157															0																						
162																												1	1	0				3			
163													4	2	0															0				14			
166			7																																		
175	**						16																		9		24			24							
186																0	1											0									
206									0		1		16				0	12				13			0												
209													28			10	0											4									
219	**			27				13					35		8	25												0									
222																	0	0		8								0									
232																		2																			
240									22															0													
242	*									23											22				6				15								1
247															0																						
253			20		8								30					0						3													

f)	Year	1981	1982	1983	1984	1985	1986	1987	1988	1989	1990	1991	1992	1993	1994	1995	1996	1997	1998	1999	2000	2001	2002	2003	2004	2005	2006	2007	2008	2009	2010	2011	2012	2013	2014	2015	2016
Country																													*								
12	**									21								8			13						5										
14			1												1								0				0	43									
15																		0										2									
21					0												3		0	2	2			3													
27				9		1						0						0	12																		
30							7																	1	0												
32			7								0	4				1		3	0																		
36				5													11	0		0								10									
41									5														2								0						
48								0			0					0																					
52			2		0																1	0															
68													0		0						1		0													0	
72																	24				0		1						2							1	
74		7														8					0		33													0	
79											9		0																		2	13					1
85					2										4					5	12			4						0							
89						7					7									1		1		0				2									
103																	0			0	16			20				6									
107	**									19																			19								
108					8													0		9				0				0	19								
112									1	0								9				0		13				1									
117	**											15							16												24						
118					5									1																0					3		2
135								15													13	0															
147	*			1													12			3	11			16				20									
149								6																				6									
153						0	0	6														0		1													
166											0						9				16															22	
176											5	1							0		0																
179																			0	2	1						5										
185				0													7				20			20				8									
186																			1																		
205																	0			0	9			17													
206			2								1			14									2	16				5									
208									5																					2							
209				5			0			0			6			2								0						1							
212																0				0																	
216		0																											2							6	
222										23																			19						1		
225	*																				22									8							
232																												0									
236														21							7									21						0	3
237																	4			0	0			7													
240									15														0														
243														0	0				0											0							
253																																		0	0		20

g)	Year	1981	1982	1983	1984	1985	1986	1987	1988	1989	1990	1991	1992	1993	1994	1995	1996	1997	1998	1999	2000	2001	2002	2003	2004	2005	2006	2007	2008	2009	2010	2011	2012	2013	2014	2015	2016
Country										*														*					*								
1																	0																				
3												1					2							9													
4	**																				32		29						26								
11																													25								
12													10																	16	2						
19																										0	4	0									
21																			0	2	2	6									0						
22				1				1																													1
27											7						4				4							0									
29																				0		1		38							0				2		
30																								1													
32			61																		34		0				10										
36						29											8							11				44									
41									49	3					3							40	57	5													
47				2																									6	3							
57																	3			0				37							1						
61														24					11		10			24			12										
63							9	5					25															2				9					
69																															19						
74															12					4										0							
79			5	0			21																					1			4	27					4
85															11				10		23									21	1						
86			14	6																				29								16					
89																								1				20									
103														32			2							33													
107																													35					0			
108																					34	3				0											
109						2	16	10						18										5						11							
111										12																			19								
112																						0		12													
114																12	5													6	0						
116			7				26																				5	0	20								
117			24													32																					
118															8		0	17		12	0								0								
120																	6							12				26									
122	**		53													29																	4		41		
123											3	5				17																					
124														12	12	0				19							64					10					
125								9		50								3		43									41		9				37		0
126												14																	0								
128																					3																
130														9	6	3				12							65				1	12					
131			5	4		16		2																									11			13	3
133																	5					6		10				22									
147	**																10				13			22				57					40				
150													13				4			0	26			35													
152																				8	22					17		3									
153																								0													
154											0	1				0	0		2																		
157													6	0																							
158				6		14		0																				2									
166																		9			10													0	19	28	2
172						6									0			0		30	19								13								
175															0				0	7	0														2		
176																												0									
179													17		6			29			1			37			23										
180	*			22												15				22						35											
185	**																8						43	39				46									
186																1			1		0										7						
198														0																							
200																	4				23			33				33									
205																					5			28							1						
206							3																	38				33		4							
209																								0													
211					10					20				0							31																
212															4					0										0	1						
213	*															23										35											
216																				0										19			16				
219													40										3	0	0	2					13						
220						2		5																							0						
221			7	2			15	7																							15						8
222	**									35																			37						23		
224															0	1	25			0	47	45															
226									0								0		0	0	0																
231										24																								14			
232																												18									
233																	48																				
236									3						12					1									7	2	0						
237																	7				0			20				41									
239								2																				2					26				
240																							9														
242														4						18		0	0														
252																											0										
253			5																					0													
