# Supplementary material for: Complex drought patterns robustly explain global yield loss for major crops
Source: Sci Rep. 2022 Apr 6;12:5792. doi: 10.1038/s41598-022-09611-0 (PMC8986840; doi:10.1038/s41598-022-09611-0)
Supplement: Supplementary file 3 — Supplementary Information 3. [file 41598_2022_9611_MOESM3_ESM.pdf]

	Year	1981	1982	1983	1984	1985	1986	1987	1988	1989	1990	1991	1992	1993	1994	1995	1996	1997	1998	1999	2000	2001	2002	2003	2004	2005	2006	2007	2008	2009	2010	2011	2012	2013	2014	2015	2016
Country					***								***								***												**				
1																	0	0	0	0	15																
3												1	2	3	0		2							9													
4	**																				32		29						26								
7										2	15			0	0	0											0	0									
11										32	0				0		8	6			0					0			25	51	0						0
12	*									21			10					8			13						5			16	2						
14			1												1								0			8	0	43									
15					6			1					42					0					1								3						
16										39																			4								
19							5	0							1						2					0	4	0			1		3			4	
21					0												3		0	4	4	6		3							0						
22				1				1								23	29																			11	6
23						16			0	0						1																1		0	9	21	
24				18	26									0	0		1				0				0				0	1	0			29		24	16
26		8	0	0		0	5	13	1				0		0						0		2		10					23	0				15	14	4
27				9		2					7	0	0		0		4	0	12	2	4				0		1	0	0								
29									3											0	34	1		71				12			0		17		2		
30					6	5	7	15					57	7		4			1	0				8	0			11									
32			68								0	4			0	1		3	0		34		0		0	4	13										0
35								9	0																			2								1	34
36				5		29				0	1	0	1	0			30	0	2	0	24	0		11				67									
37				8	15			3																				2				10					
38																			0					4		15											
39													2	32	29			25	2				3	7	9						3					0	
40		0	0	9	25								19	0	1								0	0	1	0		0		5					8		
41				0					71	3			2		3						1	76	67	5							0		4				
45			2	10	18																								13	0							
46			0	1	10	3								7								6	13		0												
47			27	16														5	0									0	6	3	0						
48		0	0					0	2	0	0					0		0										0									
52			2		0												0	11	0	0	1	0			0	0	0				10	0		8			
55			18	0							0	0					0			2		6		0	0				0			7	24	10	0	1	
56							0	11	1	6			11		1	0		5														0			0	32	14
57																	3			0	34			70				8			1		14				
58												0	21	30	7	0	0		18							10				38	2	23				0	
61	**													24					11		10			24			12										
62						0								0	31																						
63							9	5					25															2				9					
66									2				1		6			15	3		17	2			7	0	0			0							
68							6						0		0		14		0	1	1		8													0	
69																															19					0	0
70																	0		0	0	0		4	0	3	0	0				7		4				
71		0			0	3	0			1						1		0					0	0	0	0			0					0	4		0
72												0					24				0		1	0	0				4							1	
74		7													13	8		4		4	0		33	5	14	0	13	0	0	1						0	
79	*		5	0	6		21			18	26		0											37				1			6	40		1		3	40
80			2	7																	0					1	0										
83																							15		9												
85					2										15				12	5	49	0		4						21	1						
86			14	6	2																			54			5				1	16				5	
87			4	29	26		0			2			3						12									0									
89			0	1		7					7	0			0				4	1		1		1				22									
94						0		0									1	4	0					19	10	2	0	0									
96								0	2	0				0															0	0				0	3	3	1
97		0	3	23																0		4	23		14						3	11		0	12	12	6
98				3					4		0			9														0	0		2						
99								0	0		28	12	3	6	4				0			4	13		8							4	6				
101			6									15							0	0	2		5		0	0					2						
103	**										6		18	54	1		2			0	49			83				12					26				
105								13							0						11															4	
106																		0								0					0						
107										19																			54			0	0	0			
108					8													0		24	52	3		0		0		0	19								
109	*					2	16	10						18										5						11							
111			6	6			9			12																			19						1	0	
112				11					1	0								9			0	0		83				1					0				
114																12	5							1				0		6	0						0
116			7				26																				5	0	20								
117			24	0			3					15			0	40	0	5	16			16		1	2	4					24			1			
118					46									1	8		5	43		12	0								2	7					3		2
120	**								12		20		3	24			6							12			2	31			1		17				
122			53												5	39	0	0															4		41		
123										4	4	5			0	17						1						0	0							18	3
124	*													12	12	0				19							64					10					
125	**							9		50								3		43									41		9				37		0
126			12					12				14	54		1	44	0											4	0								
127							0	0	2	3		3	3										0	0	0							0			0	20	
128																					3																
130														9	6	3				12							65				1	12					
131			5	4		16		2																									11			13	3
133	**													13			5				20	9		10				22			1						
134													0						4	0	11	4	0	0								0					
135								15					56								13	0			0	12											0
136					0	0		2	0									3												0					0	0	11
138					22				0	0	0		0	0	0	0	0					4	21		0	0				3		18					
142				12					0		0			0	0	0	0						12				1										
145		0								3						0	0							0	0	0	0	1	1		1						
147	***			1									8		8		22			3	39			38				101					44				
149				0				6			0		0						0									14	0					0			
150	***								3		20		16	14			4			0	56			65				17					22				
152														0		7				11	22	19				33		3									
153			4			0	0	17				5	92		38		0					0		1	0	6								0			
154			0								0	1			0	0	0		15	0	0															8	
155													20				29		21	7	0			0	0												
157			2				0						6	0	0																						
158				6		14		0																				2									
161		0	9	11								0							0	0		15	2	0		0	0									5	
162					17	0					0	0																1	1	0	0	3		3			
163					26	0						0	9	2	1						15	0	0		0			0		0				19			
166			7								0					1	17	33	0	0	50							5					1	0	19	50	2
170																					7	4	16	0													
172						6									0			0		30	19								13								
173			0	0								0				1	0	0		0		0	3	0	0	0							0				
175							16								0				0	7	32		0		9	24	34	2		56					2		4
176											5	1	29		0	4		0	0	0	0							0									
177																	2		20	0	0		0														
179	*												17		6			29	0	2	2			37			28										
180	***			22									16	2		15		7		22						35				20							
183			1								0		32	0	10	0									17												1
185	**			0									2				15				40		43	59				66					4				
186																1	1	1	2		0	0		3				0			7						
187																		5	0					7		20		2	3								
198														0				0	1	1																	
199															0	0		5	3				39				0	1				3					
200	***										16		7	23			4				59			62				41					20				
202							8														0	0	0	20	0	20										0	0
205											7		22	24	1		0			0	36			74				6			1						
206			2				3		0		2		34	14			0	12				13	2	54	0			38		4				17			
208			5						5						12			16	0								0		0	2							
209				5			0			0			64			15	0		4	5				0				25		1							
211		3			10					20		1		0					0	0	43		2	0				0									
212															4	0	0	0	0	0	0			0			0			0	1						
213														1		23										35											
215					5										0					6	0	4	17	0		0	1	1									
216		0																		0									2	19			16	0		6	
217																										2											
219	*			62				13					121		8	25							13	1	0	2		0			13						
220						2		5																							0						
221			7	2	17		15	7								0								27							18			0		14	10
222										58							0	0		8								0	56						24	0	
224															0	3	25			0	59	45															
225			3										3		16			25	0		22			3		0			3	8							
226									0					23			0		0	0	0															12	14
227							0	16											7															11	0	22	1
231										24																								14			
232																2		2				4	0					18									
233						2											48	0	0	0		18	0				2										
236									3					21	12		0	4		1	7				4	0	14	0	7	23	0					0	3
237													9				11			0	1			27				51					5				
239								2																				2					26				
240				0					41										0	14			9	0									0				
242										23	0			4	0				0	18	54	0	0	0	6	0	0		15	29		28					1
243														0	0				0	0	12	14								0							
246						0	1	0	3																											53	6
247															0	0													0								0
251																			0	0								0									
252													46						4	0		0	7			8	0										
253			25	23	16			24					57			9		0					8	3		1			4					0	0		20
